# Assessment of a method to detect signals for updating systematic reviews

**DOI:** 10.1186/2046-4053-3-13

**Published:** 2014-02-14

**Authors:** Paul G Shekelle, Aneesa Motala, Breanne Johnsen, Sydne J Newberry

**Affiliations:** 1 RAND Corporation, RAND Health, 1776 Main Street, Santa Monica, CA 90407, USA; 2 West Los Angeles Veterans Affairs Medical Center, 11301 Wilshire Blvd., Los Angeles, CA 90073, USA

**Keywords:** Methods, Systematic reviews, Updating

## Abstract

**Background:**

Systematic reviews are a cornerstone of evidence-based medicine but are useful only if up-to-date. Methods for detecting signals of when a systematic review needs updating have face validity, but no proposed method has had an assessment of predictive validity performed.

**Methods:**

The AHRQ Comparative Effectiveness Review program had produced 13 comparative effectiveness reviews (CERs), a subcategory of systematic reviews, by 2009, 11 of which were assessed in 2009 using a surveillance system to determine the degree to which individual conclusions were out of date and to assign a priority for updating each report. Four CERs were judged to be a high priority for updating, four CERs were judged to be medium priority for updating, and three CERs were judged to be low priority for updating. AHRQ then commissioned full update reviews for 9 of these 11 CERs. Where possible, we matched the original conclusions with their corresponding conclusions in the update reports, and compared the congruence between these pairs with our original predictions about which conclusions in each CER remained valid. We then classified the concordance of each pair as good, fair, or poor. We also made a summary determination of the priority for updating each CER based on the actual changes in conclusions in the updated report, and compared these determinations with the earlier assessments of priority.

**Results:**

The 9 CERs included 149 individual conclusions, 84% with matches in the update reports. Across reports, 83% of matched conclusions had good concordance, and 99% had good or fair concordance. The one instance of poor concordance was partially attributable to the publication of new evidence after the surveillance signal searches had been done. Both CERs originally judged as being low priority for updating had no substantive changes to their conclusions in the actual updated report. The agreement on overall priority for updating between prediction and actual changes to conclusions was Kappa = 0.74.

**Conclusions:**

These results provide some support for the validity of a surveillance system for detecting signals indicating when a systematic review needs updating.

## Background

Systematic reviews are a cornerstone of evidence-based care, either by themselves or through their incorporation into practice guidelines, performance measures or other evidence-based practice. To be useful, however, systematic reviews need to be up-to-date.

The science of determining when systematic reviews need updating has been developing for the past decade. Prior to 2001, no method or criterion existed to determine whether evidence-based products remained valid or whether the evidence underlying them had been superseded by newer work. Since then, several groups have begun developing methods to determine signals for updating reviews [1-5]. Most methods involve some form of limited literature searches and the use of expert opinion, although some methods use statistical methods and are applicable only to meta-analytic results [6,7]. Two of these methods have been formally compared and found to produce similar results [2]. To date, however, no method has been assessed for predictive validity, meaning there is no way of determining whether the presence or absence of signals does in fact predict whether the review is out-of-date. In addition to the more easily assessed situation of a false-positive (that is, a signal that detects that a review is out-of-date, but the subsequent update does not result in any important changes in the conclusions), such a study requires being able to assess for false-negatives, which requires updating reviews for which no signals are detected. In 2008, we were asked to determine which of 11 systematic reviews sponsored by the Agency for Healthcare Research and Quality (AHRQ) Comparative Effectiveness Review (CER) program might be in need of updating. We took advantage of a natural experiment to assess the predictive validity of our method for assessing for signals for updating.

## Methods

In this study, we assessed the predictive validity of signals for updating CERs detected in 2009 that have since been updated. We start with a description of the original process used to detect signals [3] and then describe how we assessed the validity of the signals. This original process subsequently evolved to the process described by Ahmadzai *et al.*[8]; the two are nearly identical.

### The 2009 method for detecting signals

#### Identifying new evidence from published studies

*Search strategy*. We started by using the search strategy employed in the original report. However, we limited the search (which included at least MEDLINE/PubMed and/or Cochrane Reviews, as well as, on a topic-specific basis, additional databases) to five top-rated general interest medical journals (*Annals of Internal Medicine*, *British Medical Journal*, *Journal of the American Medical Association*, *The Lancet* and *New England Journal of Medicine*) and the specialty journals most relevant to the topic. The specialty journals were those most highly represented among the references from the original report (four to six specialty journals). We also modified the key terms if, for example, we were aware of new drugs for the condition, adding their names to the search terms. Search inception dates were 6 to 12 months prior to the end date of the original CER search in order to ensure overlap between the searches.

*Study selection and extraction*. Using the same general inclusion and exclusion criteria as the original CER, a single reviewer experienced in systematic reviews conducted a screening of the titles and abstracts and requested any articles deemed relevant to the topic. From among those articles, the reviewer extracted relevant data from articles that met the inclusion criteria and then constructed an evidence table. These data included study-level details extracted in the original CER (for example, sample size, study design, and outcomes measured) as well as the outcomes themselves.

*Identifying new evidence from experts and expert opinion.* For each topic, we created a questionnaire matrix that listed the key questions and conclusions from the original executive summary. The matrix was sent to experts in the field, including the original project leader, technical expert panel members and peer reviewers. The experts were asked to indicate whether each conclusion listed in the matrix was, to their knowledge, still valid and, if not, to describe any new evidence and provide citations.

*Assessing individual conclusions for signals*. Once abstraction of the study conditions and findings for each new included study was completed and expert opinions were received, we assessed, on a conclusion-by-conclusion basis, whether the new findings provided a signal for the need for an update. Table 1 lists the criteria used for making these determinations [9].

**Table 1:** Criteria for determining signals for updating

**Label**	**Indications for the need for an update**
Still valid	Original conclusion is still valid and this portion of the original report does not need updating. This conclusion was reached if we found no new evidence or only confirmatory evidence and all responding experts assessed the CER conclusion as still valid.
Possibly out of date	Original conclusion is possibly out of date and this portion of the original report may need updating. This conclusion was reached if we found some new evidence that might change the CER conclusion, and/or a minority of responding experts assessed the CER conclusion as having new evidence that might change the conclusion.
Probably out of date	Original conclusion is probably out of date and this portion of the original report may need updating. This conclusion was reached if we found substantial new evidence that might change the CER conclusion, and/or a majority of responding experts assessed the CER conclusion as having new evidence that might change the conclusion.
Out of date	Original conclusion is out of date. This conclusion was reached if we found new evidence that rendered the CER conclusion out of date or no longer applicable. Recognizing that our literature searches were limited, we reserved this category only for situations where a limited search would produce prima facie evidence that a conclusion was out of date, such as the withdrawal of a drug or surgical device from the market, a black box warning from FDA, etc.

For each CER, we constructed a summary table that included the following for each key question: original conclusions, findings of the new literature search, summary of expert assessment, our final assessment of the currency of the conclusions, and the priority for updating.

*Determining priority for updating a CER*. We needed to make an overall judgment regarding the priority for updating an entire CER. This determination rested on two criteria. (1) How much of the CER is possibly, probably or certainly out-of-date? (2) How out-of-date is that portion of the CER? For example, we asked whether the potential changes to the conclusions would involve only refinement of original estimates or whether the potential changes would include the finding that some therapies are no longer favored or might no longer be in use. Another question was whether the portion of the CER that was probably or certainly out-of-date involved an issue of safety (for example, a drug withdrawn from the market, a US Food and Drug Administration black box warning) or the availability of a new drug within an existing class, with the latter being a less important signal to update than the former. This final determination was a global judgment made by all the individuals working on each particular CER. On the basis of that determination, we classified CERs as being of low, medium or high priority for updating. For high-priority updates, we also provided our rationale.

### Assessment of predictive validity

Our 2009 work assessed 11 CERs. We classified four as having a high priority for updating, four as having a medium priority for updating and three as having a low priority for updating (see Table 2). One of the low-priority topics, comparative effectiveness of percutaneous coronary interventions and coronary artery bypass grafting for coronary artery disease, was considered a low priority for an update because AHRQ had already commissioned an individual patient data meta-analysis, which it considered to be an update of the CER and was published in 2009 [10].

**Table 2:** **Comparative effectiveness reviews assessed**^**a**^

**CER**	**2009 prediction**	**Update commissioned by AHRQ**
*Comparative Effectiveness of Management Strategies for Gastroesophageal Reflux Disease*[11]	High	Yes
*Effectiveness of Noninvasive Diagnostic Tests for Breast Abnormalities*[12]	High	Yes
*Comparative Effectiveness of Epoetin and Darbepoetin for Managing Anemia in Patients Undergoing Cancer Treatment*[13]	High	Yes
*Comparative Effectiveness and Safety of Analgesics for Osteoarthritis*[14]	High	Yes
*Efficacy and Comparative Effectiveness of Off-Label Use of Atypical Antipsychotics*[15]	Medium	Yes
*Comparative Effectiveness of Drug Therapy for Rheumatoid Arthritis and Psoriatic Arthritis in Adults*[16]	Medium	Yes
*Comparative Effectiveness of Treatments to Prevent Fractures in Men and Women with Low Bone Density or Osteoporosis*[17]	Medium	Yes
*Comparative Effectiveness of Second-Generation Antidepressants in the Pharmacologic Treatment of Adult Depression*[18]	Low	Yes
*Comparative Effectiveness of Angiotensin-Converting Enzyme Inhibitors (ACEIs) and Angiotensin II Receptor Antagonists (ARBs) for Treating Essential Hypertension*[19]	Low	Yes
*Comparative Effectiveness of Therapies for Clinically Localized Prostate Cancer*^b^[20]	Medium	No
*Comparative Effectiveness of Percutaneous Coronary Interventions and Coronary Artery Bypass Grafting for Coronary Artery Disease*^c^[21]	Low	No

^a^AHRQ, Agency for Healthcare Research and Quality; CER, comparative effectiveness review. ^b^Update not commissioned pending publication of the PIVOT trial. ^c^Update not commissioned or individual patient data meta-analysis had already been commissioned.

AHRQ elected to support full updates of all of the remaining CERs except the report on clinically localized prostate cancer, for which they believed it would be prudent to wait for the pending PIVOT trial results [22]. This situation presented us with a natural experiment. Because all of the reports, regardless of update priority status, were going to get the gold standard of a complete update, we could assess for both false-positives (reports classified as high priority but having no major change in conclusions when updated) and false-negatives (reports classified as low priority that, when updated, had major changes in conclusions) based on the 2009 predictions. To do this experiment, we took each conclusion from the original CER and then tried to match it with the closest similar conclusion from the update. We then assessed the degree of concordance between the 2009 prediction and the updated conclusion. We used the criteria described below.

1. Good: Concordance was considered good if the original prediction was “still valid” and there was no new relevant evidence or if new evidence continued to support the conclusion, or if the original prediction was “possibly out-of-date”, “probably out-of-date” or “out-of-date” and new evidence appeared that changed the conclusions by a substantial amount.

2. Fair: Concordance was considered fair if the original prediction was “still valid” and new evidence supported changes in some conclusions but not others or if the original prediction was “possibly out-of-date” but no new evidence was incorporated into the updated conclusions and there were no substantive changes from the original conclusions; or if the original prediction was “probably out-of-date” or “out-of-date” and some conclusions or some aspects of the conclusions had changed but others had not.

3. Poor: Concordance was considered poor if the original prediction was “still valid” but new evidence substantially changed the conclusions or if the original prediction was “probably out-of-date” or “out-of-date” but no new evidence was incorporated into the update and the conclusions underwent no substantive changes.

Examples of the degree of concordance analysis are shown in Table 3.

**Table 3:** **Examples of degree of concordance between 2009 prediction and updated conclusion**^**a**^

**Examples**	**Predictions and conclusions**
Example 1	
Original conclusion (from CER on analgesics for osteoarthritis)	No clear differences between various nonaspirin, nonselective NSAIDs or partially selective NSAIDs with regard to efficacy for pain relief or improvement
2009 surveillance assessment [14]	Conclusion still valid
Conclusion from 2011 CER update [23]	No clear difference in efficacy for pain relief, or withdrawals due to lack of efficacy
Concordance	Good
Example 2	
Original conclusion (from CER on analgesics for osteoarthritis)	Etoricoxib is associated with fewer gastrointestinal adverse events than nonselective NSAIDs
2009 surveillance assessment [14]	Possibly out-of-date
Conclusion from 2011 CER update [23]	No comparable conclusion, as etoricoxib was not included because it did not gain FDA approval for sale in the United States
Concordance	Good
Example 3	
Original conclusion (from CER on second-generation antidepressants)	Overall discontinuation rates did not differ significantly between SSRIs as a class and bupropion, mirtazapine, nefazodone, trazodone and venlafaxine. In the case of venlafaxine compared with SSRIs, higher discontinuation rates due to adverse events appeared to be balanced by lower discontinuation rates due to lack of efficacy.
2009 surveillance assessment [16]	Conclusion is possibly out-of-date, and this portion may need updating based on new analysis showing lower dropout rate with escitalopram.
Conclusion from 2011 CER update [24]	Meta-analyses of numerous efficacy trials indicate that overall discontinuation rates are similar. Duloxetine and venlafaxine have a higher rate of discontinuations due to adverse events than SSRIs as a class. Venlafaxine has a lower rate of discontinuations due to lack of efficacy than SSRIs as a class.
Concordance	Fair: Escitalopram data did not end up in the conclusions
Example 4	
Original conclusion from CER on second-generation antidepressants	Three head-to-head RCTs suggest that no substantial differences exist between fluoxetine and sertraline, fluvoxamine and sertraline, and trazodone and venlafaxine regarding relapse. Twenty-one placebo-controlled trials support the general efficacy and effectiveness of most second-generation antidepressants for preventing relapse or recurrence. No evidence exists for duloxetine.
2009 surveillance assessment [16]	Conclusion is possibly out-of-date, and this portion of the CER may need updating to include evidence for duloxetine.
Conclusion from 2011 CER update [24]	On the basis of results of six efficacy trials and one naturalistic study, no significant differences exist between escitalopram and desvenlafaxine, escitalopram and paroxetine, fluoxetine and sertraline, fluoxetine and venlafaxine, fluvoxamine and sertraline, and trazodone and venlafaxine for preventing relapse or recurrence.
Concordance	Fair: No duloxetine evidence ended up being included with regard to this key question
Example 5	
Original conclusion (from CER on management of GERD)	Medical therapy with PPIs and surgery (fundoplication) appeared to be similarly effective for improving symptoms and decreasing esophageal acid exposure.
2009 surveillance assessment [18]	Conclusion is still valid, and this portion of the CER does not need updating.
Conclusion from 2011 CER update [25]	The 2005 CER concluded that medical therapy with PPIs and antireflux surgery were similarly effective in improving GERD-related symptoms and decreasing esophageal acid exposure, although some surgical patients required ongoing medical therapy postprocedure. With the addition of long-term follow-up data (7 to 12 years) from two previously reviewed studies and results from two new RCTs, our updated review found that patients who underwent antireflux surgery experienced a greater improvement in heartburn and regurgitation at follow-up than did patients who received medical treatment alone.
Concordance	Poor: Update indicates symptoms are better with surgery

^a^CER, comparative effectiveness review; FDA, US Food and Drug Administration; GERD, gastroesophageal reflux disease; NSAID, nonsteroidal anti-inflammatory drug; PPI, proton pump inhibitor; RCT, randomized controlled trial; SSRI, selective serotonin reuptake inhibitor.

We assessed “concordance” rather than “agreement” because the matching of the original conclusions to updated conclusions was often challenging, and “agreement” implies a more direct comparison of original to updated conclusions than is always possible. For this reason, we refrained from using a 2 × 2 table to make comparisons.

We then made a summary assessment of the CER’s priority for updating, based on the updated conclusions. We used the same criteria as those in the prospective assessment: How much of the report was out-of-date and the degree to which it was out-of-date. Using the κ statistic, we compared the agreement between the original assessment of priority and the actual changes.

In the assessment of concordance of individual conclusions, an additional complicating factor was the time delay between our limited literature searches to assess for signals (2008) and the search dates of the update reports (2010 to 2012). Therefore, for conclusions with poor concordance, we reviewed whether they may have been influenced by new evidence published after the surveillance signals search.

## Results

We performed our assessment of predictive validity for nine CERs comprising 149 individual conclusions. For each CER, we present our assessment of the concordance of individual conclusions (Additional file 1) as well as a full table describing each conclusion and how it was assessed (Additional file 2). We also provide an overall table that sums up the individual conclusion assessments across all CERs (Table 4).

**Table 4:** **Summary of concordance of predicted and actual conclusions across nine comparative effectiveness reviews**^**a**^

**CER**	**Good**	**Fair**	**Poor**	**Total**
Still valid	83	1	1	85
Possibly out-of-date	11	16	0	27
Probably out-of-date	7	0	0	7
Out-of-date	4	4	0	8
Total	105	21	1	127

^a^CER, comparative effectiveness review. Not applicable/no matching conclusions/new conclusions = 22.

The great majority (83%) of conclusions for each CER and across CERs had good concordance. However, the CER on gastroesophageal reflux disease (GERD) had four “out-of-date” conclusions with only fair concordance, and one conclusion we had assessed as “still valid” was shown to be out-of-date.

The published 2009 updating assessment judged that the conclusion regarding endoscopic treatment for GERD “should be deleted”, meaning that it was out-of-date, because the endoscopic procedures had been withdrawn from the market. However, one of the three endoscopic procedures reviewed in the original report continued to be used, new endoscopic procedures were introduced and one of the two withdrawn procedures was later reintroduced. The update report noted this changing landscape, and we deemed the concordance with the 2009 prediction as only fair. A more appropriate surveillance assessment would have been that the conclusion needed updating because the endoscopic procedures were evolving over time.

Another conclusion in the original GERD report—that surgery and medical therapy were similarly effective—was rated as “still valid” during the surveillance process but had poor concordance with the update review, which concluded that surgery was favored over medical therapy. One of the studies providing new evidence in support of this conclusion was published in 2009, after completion of the surveillance signal search.

Table 5 compares our original predictions of the need for updating with the priority as determined by the actual update. One CER that was predicted in 2009 to be a high priority for updating was judged to have been a medium priority for updating based on the updated report. A CER determined to be a medium priority update was originally judged as having been a high priority for an update. The updating priority remained the same for the other seven CERs. Table 6 presents in a 3 × 3 table the results of the overall assessment of priority for updating. The κ statistic for agreement was 0.74 (Table 6).

**Table 5:** **Comparison of predicted vs. actual priority for updating**^**a**^

**CER**	**2009 prediction**	**End date of update search**	**2013 assessment**	**Rationale**
*Comparative Effectiveness of Management Strategies for Gastroesophageal Reflux Disease*[25]	High	August 2010	High	Some procedures specifically mentioned in the Executive Summary have been withdrawn from the market. New procedures have been introduced. There is a major change in the conclusion about surgery vs. medical therapy.
*Effectiveness of Noninvasive Diagnostic Tests for Breast Abnormalities*[26]	High	September 2010	Medium	The new data did not change the overall conclusions very much. The conclusion that MRI and ultrasound may be sufficient to evaluate lesions in women at low risk may be an important new conclusion.
*Comparative Effectiveness of Epoetin and Darbepoetin for Managing Anemia in Patients Undergoing Cancer Treatment*[27]	High	April 2012	High	Major safety concerns leading to substantial changes in black box warnings and practice guidelines
*Comparative Effectiveness and Safety of Analgesics for Osteoarthritis*[23]	High	January 2011	High	The updated Executive Summary specifically mentions a number of drugs that have been withdrawn because of safety concerns.
*Efficacy and Comparative Effectiveness of Off-Label Use of Atypical Antipsychotics*[28]	Medium	May 2011	Medium	There are many new off-label indications and data on effectiveness, but these do not indicate strong effects of these drugs.
*Comparative Effectiveness of Drug Therapy for Rheumatoid Arthritis and Psoriatic Arthritis in Adults*[29]	Medium	February 2011	High	New, expensive biologic DMARDs feature prominently in the Executive Summary of the updated report.
*Comparative Effectiveness of Treatments to Prevent Fractures in Men and Women with Low Bone Density or Osteoporosis*[30]	Medium	March 2011	Medium	There are two new drugs: zoledronic acid and denosumab. However, there is no evidence that they are any more effective than existing drugs. There are signals of serious but rare new side effects, in particular subtrochanteric fractures of the hip, but they are not sufficient to change the initial decision to recommend antiresorptive therapy for women with osteoporosis.
*Comparative Effectiveness of Second-Generation Antidepressants in the Pharmacologic Treatment of Adult Depression*[24]	Low	January 2011	Low	No substantive changes in conclusions
*Comparative Effectiveness of Angiotensin-Converting Enzyme Inhibitors (ACEIs) and Angiotensin II Receptor Antagonists (ARBs) for Treating Essential Hypertension*[31]	Low	December 2010	Low	No substantive changes in conclusions

^a^DMARD, disease-modifying antirheumatic drug; MRI, magnetic resonance imaging.

**Table 6:** **Predictive validity of priority for updating a systematic review (2009 predicted priority)**^**a**^

	**Priority based on actual changes in conclusions**			
**Priority**	**High- 2013 assessment**	**Medium- 2013 assessment**	**Low- 2013 assessment**	**Overall**
High- 2009 prediction	3	1	0	4
Medium- 2009 prediction	1	2	0	3
Low- 2009 prediction	0	0	2	2
**Total**	4	3	2	9

^a^κ = 0.74.

## Discussion

This assessment of the predictive validity of a method to assess a CER for signals for updating yielded generally favorable results. For the vast majority of individual conclusions, concordance between the 2009 predictions and the subsequent updated conclusions was judged to be good. The one instance of poor concordance had new evidence published after the surveillance signals had been assessed, and in this instance involved a CER already judged to be of high priority for updating based on signals of other out-of-date conclusions.

Our present study has three primary limitations. The first is sample size. We were able to assess only nine CERs. However, this number included CERs assessed as being of high, medium or low priority, thus allowing us to assess the possibility of false-negatives (that is, CERs assessed as low priority for updating that nevertheless were fully updated). The likelihood of assessing such false-negatives again is small, as it requires that low-priority CERs be subjected to the gold standard of a full update. Our findings that neither of the CERs judged to be a low priority had any substantive changes in conclusions will reinforce the decision to invest scarce resources in researching other topics rather than commisioning updates of low-priority CERs.

A second limitation is the matching of original conclusions to updated conclusions. In some updated reports, the authors themselves matched the conclusions. In most cases, however, this was not done, and, in some circumstances, determining the appropriate match to the original conclusion was challenging. Additional file 2 lists each original conclusion and its matching updated conclusion so that readers may judge this agreement for themselves.

The third principal limitation of this study is that the 2013 assessment of the 2009 predictions could not be made in a blinded fashion. Our Evidence-based Practice Center (EPC) did both assessments, and, even if some other group had done the 2013 assessment, we could not have enforced blinding, because the 2009 assessments are in the public domain. We tried to guard against bias by having explicit reasons for each judgment and presenting these reasons for readers themselves to judge. Our reasoning should be transparent.

With the limitation of small sample size in mind, we offer the following preliminary conclusions about the surveillance signal method. (1) Low-priority CERs are unlikely to have any substantive changes in conclusions. (2) Conclusions judged likely to be “still valid” almost certainly are still valid. (3) Conclusions judged to be “out-of-date” almost certainly are out-of-date. (4) Safety concerns and the appearance of new classes of therapies and more efficacious treatments are the best targets for high-priority updates. (5) The classification of individual conclusions as possibly or probably out-of-date owing to new evidence may be slightly too sensitive as a signal; in a number of such instances, the update report’s conclusion did not change, because the new evidence identified in the signal search was either rejected or insufficient to change the original conclusion.

In sum, our assessment provides some support for the predictive validity of this method of assessing CERs for signals of the need for updating. Future research is likely to be confined to assessing updates of systematic reviews judged to be a medium or high priority for updating. Further assessment of the factors leading to changes in individual conclusions may help refine the criteria for distinguishing between high- and medium-priority update topics. However, investing extra time and effort to distinguish “possibly” from “probably” out-of-date conclusions or to further refine the global assessment to distinguish medium- from high-priority update topics may begin to make the surveillance process resemble the actual update, which is not the goal of surveillance. In this application, the surveillance process worked very well—nearly perfectly, in fact (κ ≥ 0.8 is considered nearly perfect agreement). No low-priority CER was judged, as having had a substantive change to a conclusion in the update, whereas 3 of 4 high priority CERs did have substantive changes to the conclusions. The results suggest that it is very unlikely that new, practice-changing evidence exists concerning a systematic review judged to be a low priority for updating and supports a policy of delaying an update of a systematic review until new evidence is sufficient to warrant assigning it at least a medium priority.

The assessment method described herein represents part of the basis for the surveillance method used to assess AHRQ systematic reviews as described by Ahmadzai *et al*. [8]. That program was designed to assess each AHRQ systematic review every 6 months and to take 3 months to complete. One important result is that no systematic review was judged to be a high priority for updating at the first 6-month assessment, meaning that it is probably more cost-effective to assess systematic reviews no more frequently than yearly. Additional work on making surveillance more cost-effective is warranted.

## Conclusion

In our present study, we found evidence supporting the predictive validity of a method for assessing AHRQ systematic reviews regarding their need for updating. One advantage of this method relative to other proposed methods is that it is equally useful for meta-analytic reviews and narrative reviews. It may be applicable to systematic reviews produced by other organizations.

## Competing interests

The authors declare they have no competing interests.

## Authors’ contributions

PGS developed the idea for the study. PGS and SJN developed the original 2009 method and its applications. AM and BJ collected the information from the updated reports and performed the initial matching of conclusions and PGS revised these matches, made the determinations of agreement and concordance, and performed the statistical analysis. All authors read and approved the final manuscript.

## Supplementary Material

Additional file 1 **Concordance of predicted and actual conclusions for update of the nine Comparative Effectiveness Reviews.** The table presents the authors assessment of the concordance of individual conclusions for each of the nine comparative effectiveness reviews by listing the amount of conclusions from the report that that were “still valid”, “possibly out of date”, “probably out of date”, “out of date”, or were “not applicable/no matching conclusions/new conclusions” to those that were rated as “good”, “fair”, “poor”, or “not rated”. Click here for file

Additional file 2 **Conclusion assessments across all nine Comparative Effectiveness Reviews.** The table presents the nine Comparative Effectiveness Reviews conclusions for the original review, the update review, the 2009 prediction, and the concordance for each of the conclusions. Click here for file
