# Correction: Assessment of a method to detect signals for updating systematic reviews

**DOI:** 10.1186/2046-4053-3-22

**Published:** 2014-03-26

**Authors:** Paul G Shekelle, Aneesa Motala, Breanne Johnsen, Sydne J Newberry

**Affiliations:** 1 RAND Corporation, RAND Health, 1776 Main Street, Santa Monica, CA 90407, USA; 2 West Los Angeles Veterans Affairs Medical Center, 11301 Wilshire Blvd, Los Angeles, CA 90073, USA

## Correction

Following publication of our article [1], we became aware that the following disclaimer was missing:

This project was funded under Contract No. HHSA290200710062I from the Agency for Healthcare Research and Quality, U.S. Department of Health and Human Services. The authors of this report are responsible for its content. Statements in the report should not be construed as endorsement by the Agency for Healthcare Research and Quality or the U.S. Department of Health and Human Services.
