# Supplementary material for: Assessment of a method to detect signals for updating systematic reviews
Source: Syst Rev. 2014 Feb 14;3:13. doi: 10.1186/2046-4053-3-13 (PMC3937021; doi:10.1186/2046-4053-3-13)
Supplement: Additional file 1 — Concordance of predicted and actual conclusions for update of the nine Comparative Effectiveness Reviews. The table presents the authors assessment of the concordance of individual conclusions for each of the nine comparative effectiveness reviews by listing the amount of conclusions from the report that that were “still valid”, “possibly out of date”, “probably out of date”, “out of date”, or were “not applicable/no matching conclusions/new conclusions” to those that were rated as “good”, “fair”, “poor”, or “not rated”. [file 2046-4053-3-13-S1.doc]

**Additional File 1. Concordance of predicted and actual conclusions for update of the nine Comparative Effectiveness Reviews**

| **Concordance of predicted and actual conclusions for update of Effectiveness of Noninvasive Diagnostic Tests for Breast Abnormalities** | | | | |
| --- | --- | --- | --- | --- |
|  | **Good** | **Fair** | **Poor** | **Not Rated** |
| Still valid | 1 |  |  |  |
| Possibly out of date | 1 | 1 |  |  |
| Probably out of date | 1 |  |  |  |
| Out of date |  |  |  |  |
| Not applicable / No matching conclusions / New conclusions |  |  |  |  |
| **Concordance of predicted and actual conclusions for update of Comparative Effectiveness of Epoetin and Darbepoetin for Managing Anemia in Patients Undergoing Cancer Treatment** | | | | |
|  | **Good** | **Fair** | **Poor** | **Not Rated** |
| Still valid | 1 |  |  |  |
| Possibly out of date | 1 | 1 |  |  |
| Probably out of date | 2 |  |  |  |
| Out of date | 1 |  |  |  |
| Not applicable / No matching conclusions / New conclusions |  |  |  | 3 |
| **Concordance of predicted and actual conclusions for update of Comparative Effectiveness and Safety of Analgesics for Osteoarthritis** | | | | |
|  | **Good** | **Fair** | **Poor** | **Not Rated** |
| Still valid | 19 |  |  |  |
| Possibly out of date | 2 | 2 |  |  |
| Probably out of date | 1 |  |  |  |
| Out of date | 2 |  |  |  |
| Not applicable / No matching conclusions / New conclusions |  |  |  |  |
| **Concordance of predicted and actual conclusions for update of Efficacy and Comparative Effectiveness of Off-Label Use of Atypical Antipsychotics** | | | | |
|  | **Good** | **Fair** | **Poor** | **Not Rated** |
| Still valid | 16 | 1 |  |  |
| Possibly out of date | 2 |  |  |  |
| Probably out of date | 1 |  |  |  |
| Out of date |  |  |  |  |
| Not applicable / No matching conclusions / New conclusions |  |  |  | 2 |
| **Concordance of predicted and actual conclusions for update of Comparative Effectiveness of Drug Therapy for Rheumatoid Arthritis and Psoriatic Arthritis in Adults** | | | | |
|  | **Good** | **Fair** | **Poor** | **Not Rated** |
| Still valid | 11 |  |  |  |
| Possibly out of date | 2 | 2 |  |  |
| Probably out of date | 1 |  |  |  |
| Out of date | 1 |  |  |  |
| Not applicable / No matching conclusions / New conclusions |  |  |  | 2 |
| **Concordance of predicted and actual conclusions for update of Comparative Effectiveness of Treatments to Prevent Fractures in Men and Women with Low Bone Density or Osteoporosis** | | | | |
|  | **Good** | **Fair** | **Poor** | **Not Rated** |
| Still valid | 27 |  |  |  |
| Possibly out of date | 1 | 3 |  |  |
| Probably out of date |  |  |  |  |
| Out of date |  |  |  |  |
| Not applicable / No matching conclusions / New conclusions |  |  |  | 12 |
| **Concordance of predicted and actual conclusions for update of Comparative Effectiveness of Second-Generation Antidepressants in the Pharmacologic Treatment of Adult Depression** | | | | |
|  | **Good** | **Fair** | **Poor** | **Not Rated** |
| Still valid | 13 |  |  |  |
| Possibly out of date |  | 7 |  |  |
| Probably out of date |  |  |  |  |
| Out of date |  |  |  |  |
| Not applicable / No matching conclusions / New conclusions |  |  |  | 2 |
| **Concordance of predicted and actual conclusions for update of Comparative Effectiveness of Angiotensin-Converting Enzyme Inhibitors (ACEIs) and Angiotensin II Receptor Antagonists (ARBs) for Treating Essential Hypertension** | | | | |
|  | **Good** | **Fair** | **Poor** | **Not Rated** |
| Still valid | 7 |  |  |  |
| Possibly out of date | 1 |  |  |  |
| Probably out of date |  |  |  |  |
| Out of date |  |  |  |  |
| Not applicable / No matching conclusions / New conclusions |  |  |  |  |
| **Concordance of predicted and actual conclusions for update of Comparative Effectiveness of Management for Gastoesophageal Reflux Disease (GERD)** | | | | |
|  | **Good** | **Fair** | **Poor** | **Not Rated** |
| Still valid | 4 |  | 1 |  |
| Possibly out of date | 1 |  |  |  |
| Probably out of date | 1 |  |  |  |
| Out of date |  | 4 |  |  |
| Not applicable / No matching conclusions / New conclusions |  |  |  | 2 |
