# Supplementary material for: Assessment of a method to detect signals for updating systematic reviews
Source: Syst Rev. 2014 Feb 14;3:13. doi: 10.1186/2046-4053-3-13 (PMC3937021; doi:10.1186/2046-4053-3-13)
Supplement: Additional file 2 — Conclusion assessments across all nine Comparative Effectiveness Reviews. The table presents the nine Comparative Effectiveness Reviews conclusions for the original review, the update review, the 2009 prediction, and the concordance for each of the conclusions. [file 2046-4053-3-13-S2.docx]

**Additional File 2**

Additional File 2. Conclusion assessments across all nine Comparative Effectiveness Reviews

| **Original Key Questions/Conclusions** | | **Updated Key Questions/Conclusions (2011-2012)** | **2009 Prediction** | **Concordance** |
| --- | --- | --- | --- | --- |
| **Comparative Effectiveness of Management for Gastoesophageal Reflux Disease (GERD) (Original report date - Dec 2005 and Update report date - Sep 2011)** | | | | |
| **Key Question 1 - What is the evidence of the comparative effectiveness of medical, surgical, and endoscopic treatments for improving objective and subjective outcomes in patients with chronic GERD? Is there evidence that effectiveness varies by specific techniques/procedures or medications? Objective outcomes include esophagitis healing, ambulatory pH, other indicators of reflux, need for medication, healthcare utilization, and incidence of esophageal stricture, Barrett's esophagus, or esophageal adenocarcinoma. Subjective outcomes include symptom frequency and severity, sleep/productivity, and overall quality of life.** | | **Key Question 1 - What is the evidence of the comparative effectiveness of medical, surgical and other newer forms of treatments for improving objective and subjective outcomes in patients with chronic gastroesophageal reflux disease (GERD)? Is there evidence that effectiveness varies by specific technique, procedure, or medication? Objective outcomes addressed include esophagitis healing, ambulatory pH, other indicators of reflux, need for medication, healthcare utilization, and incidence of esophageal stricture, Barrett's esophagus or esophageal 4 adenocarcinoma. Subjective outcomes include symptom frequency and severity, sleep/productivity, and overall quality of life.** |  |  |
| Medical therapy with PPIs and surgery (fundoplication) appeared to be similarly effective for improving symptoms and decreasing esophageal acid exposure. 10 percent to 65 percent of surgical patients still require medications. The limited data available did not support a significant benefit of fundoplication compared with medical therapy for preventing Barrett's esophagus or esophageal adenocarcinoma. | | The 2005 CER concluded that medical therapy with PPIs and antireflux surgery were similarly effective in improving GERD-related symptoms and decreasing esophageal acid exposure, although some surgical patients required ongoing medical therapy post procedure. With the addition of long-term followup data (7 to 12 years) from two previously reviewed studies and results from two new RCTs, our updated review found that patients who underwent antireflux surgery experienced a greater improvement in heartburn and regurgitation at followup than patients who received medical treatment alone. However, some uncertainty remains in the true estimates of the efficacy of surgery versus medical treatment because of the large proportion of patient dropouts (33 to 58 percent) in studies with long followup. As with the 2005 CER, the studies in this review included patient populations with varying clinical characteristics and response to medical treatments at baseline. One of the previously reviewed studies with longterm followup data enrolled only patients with baseline esophagitis, without restriction on the degree of severity, while the other included patients with no higher than Los Angeles grade Besophagitis at randomization. | Conclusion is still valid and this portion of the CER does not need updating. | Poor - update indicates symptoms are better with surgery. However, 5 of the 8 studies contributing evidence to the update conclusion were published in 2008/2009, and the limited literature search used for the identification of signals was done in 2008 and hence certainly was not capable of detecting some of this new evidence. |
| Of the three nonrandomized studies that compared an endoscopic procedure with laparoscopic fundoplication in patients with GERD documented by pH or endoscopy, the longest follow-up was 8 months, and all three studies had significant bias that may invalidate the results. Two studies reported that more patients treated with laparoscopic fundoplication were satisfied with their results compared with those who had EndoCinchTM. One of these studies and a study of Stretta® also found less need for PPIs in patients who had fundoplication. | | Based on analysis of 4 RCTs and 3 nonrandomized trials with varied: Medical (PPI and/or H2RA) vs. surgical (open and/or laparoscopic fundoplication) interventions. Outcomes of study (GERD symptoms, QoL, satisfaction, medication use, pH study results, remission rates) Follow-up time period (1 to 12 years)Study quality (5 B-level, 2 C-level) Dropout rate for studies with 7 to 12 year followup (33 to 58%). Patients who underwent antireflux fundoplication surgery experienced a greater improvement in heartburn and regurgitation at followup compared to patients who received medical treatment alone. Surgery was associated with an increased incidence of dysphagia and postprandial bloating. Surgery decreased, but did not eliminate, the use of antireflux medications at followup. | Original conclusion should probably be deleted as the endoscopic procedure is no longer in use. | Fair - Two of the three considered endoscopic procedures had been withdrawn at the time of the 2009 surveillance, but since then 1 was reintroduced, and another was developed about 2007. The 2009 prediction probably should have been more nuanced than saying the whole conclusion should be deleted since the procedures were withdrawn, since that did not anticipate the development of new procedures. But it worked well as a signal that the procedural landscape for GERD was changing and needed updating. |
| There was no head-to-head comparison of medical treatments with endoscopic treatments. | | No study was identified for this comparison (medical vs endoscopic treatments). One small non-randomized study reported significantly better improvement in heartburn score and 24-hour pH study in the laparoscopic total fundoplication group, compared with EndoCinchTM. There were no significant differences in other outcomes. | Original conclusion should probably be deleted as the endoscopic procedure is no longer in use. | Fair - Same as above |
| PPIs were superior to H2RAs (histamine 2 receptor inhibitors) in resolution of GERD symptoms at 4 weeks and healing of esophagitis at 8 weeks.There was no difference between omeprazole, lansoprazole, pantoprazole, and rabeprazole for relief of symptoms at 8 weeks.No significant difference was found in the comparisons of esomeprazole 40 mg with lansoprazole 30 mg or pantoprazole 40 mg for relief of symptoms at 4 weeks. Similarly, there was no difference in the comparison of esomeprazole 20 mg with omeprazole 20 mg in relief of symptoms at 4 weeks. | | PPIs (esomeprazole 20 mg taken once daily or on demand, lansoprazole 15 mg taken once daily and omeprazole 20 mg taken once daily) were superior to H2RAs (ranitidine 150 mg and famotidine 20 mg, both taken twice daily) for resolution of GERD symptoms at 6 months. Data from one RCT reported that lansoprazole 15 mg, taken once daily, was more effective than ranitidine 150 mg taken twice daily for healing of esophagitis at 1 year.Data from one RCT reported that esomeprazole 20 mg, taken once daily or on demand, was more effective than ranitidine 150 mg taken twice daily for prevention of symptom relapse at 6 months.Data from two RCTs reported that maintenance treatment (≥ 6 months) with PPIs (esomeprazole 20 mg taken once daily or on demand, lansoprazole 15 mg taken once daily) appears to be more efficacious than maintenance treatment with H2RA (ranitidine 150 mg taken twice daily) in symptom remission.  Data from one RCT reported that maintenance treatment, patients taking lansoprazole 15 mg are likely to stay longer on their treatment as compared to ranitidine 150 mg taken twice daily and thus tend to have a longer median time to relapse of symptoms. Studies with larger sample sizes suggested PPIs to be more efficacious than H2RAs with respect to GERD symptoms. Based on analysis of 10 RCTs, no consistent comparative difference in symptom relief and esophagitis healing rates was observed between esomeprazole (20 to 40 mg), lansoprazole (15 to 30 mg), pantoprazole (20 to 40 mg) or rabeprazole (10to 20 mg) over a period ranging from 4 weeks to 6 months.  Data from one RCT reported that maintenance treatment, patients taking lansoprazole 15 mg are likely to stay longer on their treatment as compared to ranitidine 150 mg taken twice daily and thus tend to have a longer median time to relapse of symptoms. Studies with larger sample sizes suggested PPIs to be more efficacious than H2RAs with respect to GERD symptoms. Based on analysis of 10 RCTs, no consistent comparative difference in symptom relief and esophagitis healing rates was observed between esomeprazole (20 to 40 mg), lansoprazole (15 to 30 mg), pantoprazole (20 to 40 mg) orrabeprazole (10to 20 mg) over a period ranging from 4 weeks to 6 months.  There is some evidence from individual studies that rabeprazole 10 mg may provide better symptom relief than esomeprazole 40 mg at 4 weeks, and also that pantoprazole 20 mg provides better control of heartburn than esomeprazole 40 mg over 24 weeks. Results from three acute treatment trials showed similar esophagitis healing rates for both pantoprazole 40 mg and esomeprazole 40 mg as demonstrated by endoscopy, with the rates increasing with trial duration from 8 to 12 weeks, and being equivalent over 6 months. Based on analysis of 12 RCTs, no consistent difference in doses and dosing regiments with different PPIs in relation to symptom resolution and esophagitis healing rates. One RCT reported that there was no significant difference in symptom resolution rates at 4 weeks between esomeprazole 20 mg taken once a day and esomeprazole 40 mg taken once a day. One RCT reported a significantly higher rate of healing of esophagitis at 4 weeks was observed with esomeprazole 40 mg once a day compared with esomeprazole 20 mg once a day.  Three RCTs comparing continuous daily intake of esomeprazole 20 mg appears to provide better symptom control and quality of life relative to on demand dosing over a period of 6 months.One RCT reported that continuous daily intake of esomeprazole 20 mg appears to provide significantly better endoscopic remissioncompared with on-demand dosing over a period of 6 months. Two RCTs reported that continuous daily intake of rabeprazole 20 mg appears to provide better symptom control and quality of life relative. Based on analysis of eight RCTs, no consistent comparative difference in symptom relief and esophagitis healing rates was observed between esomeprazole (20 to 40 mg), lansoprazole 30 mg, pantoprazole 40 mg or rabeprazole 20 mg with omeprazole 20 mg or lansoprazole 15 mg over a period ranging from 4 weeks to 1 year.  One RCT reported that pantoprazole 40 mg and rabeprazole 20 mg provide significantly better symptom relief and healing of esophagitis than omeprazole 20 mg at 8 weeks. One RCT reported that esomeprazole 20 mg provides higher endoscopic remission rates compared with lansoprazole 15 mg over 6 months. | Conclusion is probably out of date and this portion of the CER may need updating based on a wealth of new data. | Good - Even though the overall conclusion didn't change, the reason for the 2009 conclusion - a wealth of new data-was supported by the update, with many of the same studies being noted. |
| For maintenance medical treatment of 6 months to 1 year, PPIs taken at a standard dose were more effective than those taken at a lower dose. | | No comparable conclusion in the update | Conclusion is still valid and this portion of the CER does not need updating. | Not applicable as there is no comparable conclusion in the update |
| Laparoscopic fundoplication was as effective as open fundoplication for relieving heartburn and regurgitation, improving quality of life, and decreasing use of antisecretory medications. Almost 90 percent of patients who were followed for 5 or more years in both surgical arms reported improvement in symptoms. | | Two RCTs and two non-randomized comparative studies compared laparoscopic fundoplication with vs. without division of short gastric vessel. No significant differences in medication use, GERD symptoms, or quality of life were found between groups. Two RCTs and one non-randomized comparative study compared laparoscopic vs. open fundoplication. No significant differences in medication use, GERD symptoms, diagnostic test results, or quality of life were found between groups. One RCT and five non-randomized comparative studies compared laparoscopic total vs. partial fundoplication. No consistent significant differences in GERD symptoms, diagnostic test results, or quality of life were observed between groups. | Conclusion is still valid and this portion of the CER does not need updating. | Good |
| Compared to sham, StrettaTM was more effective in improving symptoms of reflux and improving quality of life at 6 months and was associated with a decrease in the need for antisecretory medications. Improvement of esophageal pH exposure compared with sham could not be demonstrated for StrettaTM. | One sham-controlled study and seven noncomparative cohort studies evaluated Stretta™. In the RCT, the proportion of patients who stopped or decreased PPI use was significantly greater in the Stretta™ group compared with the control group at 6 months (but it was not significant at 1 year). No significant differences in heartburn symptoms, QoL,acid exposure and esophagitis outcomes were found. The majority of cohort studies found significant improvements in GERD symptoms, QoL, and medication use. Two sham-controlled studies and six noncomparative cohort studies evaluated the effectiveness of EndoCinch™. No consistent differences between EndoCinch™ and sham were reported. Significant improvements in heartburn, quality of life, and esophagitis healing were found in some but not all cohort studies. Five small cohort studies evaluated the effectiveness of EsophyX™.The reported proportion of patients who were off PPI at the end of the followup period ranged from 47 to 71 percent. Significant improvement of GERD-HRQL was reported by two of five studies. | Original conclusion should probably be deleted as the endoscopic procedure is no longer in use. | Fair - Same rationale as above |  |
|  | A systematic review did not find consistent effects ofPPI or H2RA (vs. placebo) in improving asthma symptoms, nocturnal asthma, use of asthma medications or FEV1. 8 primary RCTs in the update to the systematic review also reported inconsistent effects.Omeprazole 20 mg (combined with domperidone 10 mg) or esomeprazole 40 mg showed an improvement in peak expiratory flow rate. Lansoprazole 30 mg or pantoprazole 40 mg did not show an improvement in asthma symptoms or lung function tests. Rabeprazole 20 mg twice a day improved respiratory symptoms during exercise in patients with exercise induced asthma, as compared to a placebo, but not QoL or pulmonary functionmeasures. Four of six RCTs did not find a significant differencein resolution of hoarseness between PPI andplacebo.  Meta-analysis of 4 studies (191 participants) showed no significant difference in total resolution of cough between PPIs and placebo, odds ratio 0.46 (95% CI: 0.19 to 1.15). A meta-analysis of data from 4 RCTs reporting mean cough scores at the end of the trial in 109 participants found a borderline significant improvement in the mean cough scores at the end of the trial with PPIs as compared to placebo 0.38 units (95 percent CI: 0.77 to 0.00, P=0.05). Another meta-analysis examining the improvement in cough scores within the same systematic review, however, showed a significant improvement in cough scores from baseline favoring PPIs compared to placebo ( 0.39 standardized mean difference units; 95 percent CI: 0.71 to -0.08). All of the data on surgical treatment are from cohort studies, with a wide variation in the population treated, the severity of the underlying GERD and its extra esophageal manifestation, the outcome measures, the surgical interventions, the intensity and duration of followup. The majority of the cohort studies found that surgery may help improve cough and laryngeal symptoms more so than asthma, but there is a wide range of effect estimates in these studies. |  | These conclusions have no counterpart in the original report, and present new data. |  |
| **Key Question 2 - Is there evidence that effectiveness of medical, surgical, and endoscopic treatments varies for specific patient subgroups? What are the characteristics of patients who have undergone these therapies, including the nature of previous medical therapy, severity of symptoms, age, sex, weight, other demographic and medical factors, or by specific patient subgroups, and provider characteristics for procedures including provider volume and setting (eg, academic versus community)?** | **Key Question 2 - Is there evidence that effectiveness of medical, surgical and newer forms of treatments vary for specific patient subgroups? What are the characteristics of patients who have undergone these therapies, including the nature of previous medical therapy, severity of symptoms, age, sex, weight, and other demographic and medical factors? What are the provider characteristics for procedures including provider volume and setting (e.g., academic vs. community)?** |  |  |  |
| Patients on maintenance antireflux medications may have higher rates of esophagitis if they have any of the following factors: increased severity of esophagitis at baseline (pretreatment), younger age, and moderate to severe regurgitation. | One study found that there was no significant difference in the effectiveness of medical vs. surgical treatment between patients with and without Barrett’s esophagus. Six RCTs comparing different PPIs, or dosages and dosing regimens of PPIs showed mixed findings regarding the impacts of esophagitis severity at baseline on healing rates. Ten cohort studies examined patient characteristics or clinical factors as modifying factors of medical treatment outcomes. Sex was not a significant modifying factor of medical treatment outcomes. Obesity, presence of baseline typical GERD symptoms, and more severe esophagitis were significantly associated with worse medical treatment outcomes. The associations between age and medical treatment outcomes were inconsistent. The 2005 CER identified a number of patient characteristics and baseline clinical factors that may influence the effectiveness of medical, surgical, or endoscopic treatment. However, the quality and consistency of these primary data were mixed and the strength of the identified associations remained unclear. The studies included in this update were plagued with similar methodological issues. | Conclusion is still valid and this portion of the CER does not need updating. | Good |  |
| There is no substantial evidence to support a difference in surgical outcome based on age, preoperative presence or severity of esophagitis, lower esophageal sphincter incompetence, or esophageal body hypomotility. Patients treated surgically who have a history of psychiatric disorders may have worse symptom and satisfaction outcomes than those without a significant psychiatric history. | One RCT found that preoperative esophageal motility did not significantly impact the effect of laparoscopic fundoplication on dysphagia, recurrence of reflux, and acid exposure and manometry outcomes. Thirty cohort studies showed the following were inconsistently associated with worse surgical outcome: per year increase in patient’s age, morbid obesity, female sex, presence of baseline symptoms or esophagitis, and hiatal hernia greater than 3 cmat baseline. Three cohort studies examined different modifying factors of endoscopic treatment: One study did not find a significant difference between men and women in symptom improvement. One study found more patients with less severe esophagitis at baseline stopped PPI use than patients with more severe esophagitis. One study observed a learning curve in performance of a new endoscopic treatment device (EsophyX) comparing the technical procedure parameters. | Conclusion is still valid and this portion of the CER does not need updating. | Good |  |
| **Key Question 3 - What are the short- and long-term adverse effects associated with specific medical, surgical, and endoscopic therapies for GERD? Does the incidence of adverse effects vary with duration of follow-up, specific surgical intervention, or patient characteristics?** | **Key Question 3 - What are the short-term and long-term adverse events associated with specific medical, surgical, and other, newer forms of therapies for GERD? Does the incidence of adverse events vary with duration of follow-up, specific surgical intervention, or patient characteristics?** |  |  |  |
| Higher adverse event rates were described for PPIs than for H2RAs or placebo. The most commonly cited events for PPIs and H2RAs were headache, diarrhea, and abdominal pain. | One RCT reported that the rate of serious adverse events was higher in patients who underwent fundoplication than in those who had medical treatment (P = 0.06). Adverse events reported with PPIs included diarrhea, nausea or vomiting, abdominal pain, dyspepsia, and headache. These occurred in fewer than 2 percent of patients. Potential serious complications possibly associated with PPI use that were reported in the 2005 CER included enteric infections(Campylobacter and Clostridium difficile) and pneumonia. An increased risk of bone fracture is now added to this list, although the strength of association is uncertain. Common adverse events reported in patients who underwent fundoplication included bloating (up to 85 percent) and dysphagia (up to 23 percent). Reoperation rates ranged from 3 to 35 percent. Common adverse events after endoscopic suturing included chest or abdominal pain (up to 24 percent), bleeding (up to 11 percent), dysphagia (up to 50 percent), and bloating (up to 19 percent). None of these quantitative estimates are reliable because of the lack of a standard definition and uniform system of reporting. The strength of evidence was rated low. | Conclusion is possibly out of date and this portion of the CER may need updating based on expert opinion about newly recognized adverse events. | Good - the new AE of bone fracture is an important conclusion |  |
| The most commonly reported complications occurring intra operatively or within 30 days after open fundoplication were the need for splenectomy, dysphagia, inability to belch, and inability to vomit. The most commonly reported complications for laparoscopic procedures were gastric or esophageal injury or perforation, splenic injury or splenectomy, pneumothorax, bleeding, pneumonia, fever, wound infections, bloating, and dysphagia. Major complications were generally reported at very low rates. | Common adverse events reported in patients who underwent fundoplication included bloating and dysphagia. | Conclusion is still valid and this portion of the CER does not need updating. | Good |  |
| Frequently reported complications for endoscopic treatments (intra operatively or within 30 days after the procedure) included chest or retrosternal pain, gastrointestinal injury, bleeding, and short-term dysphagia. The frequency and types of complications varied with the different procedures. Serious complications, including fatalities, have also been described. | Common adverse events after endoscopic suturing included chest or abdominal pain, bleeding, dysphagia, and bloating. | Original conclusion should probably be deleted as the endoscopic procedure is no longer in use. | Fair - Same rationale as above for other conclusions about endoscopy procedures. |  |

| **Original Key Questions/Conclusions** | **Updated Key Questions/Conclusions (2011-2012)** | **2009 Prediction** | **Concordance** |
| --- | --- | --- | --- |
| **Effectiveness of Noninvasive Diagnostic Tests for Breast Abnormalities (Original report date - Feb 2006 and Update report date - Feb 2012)** | | | |
| **Key Question 1 - For the following diagnostic tests as applied to the breast (positron emission tomography (PET) scanning, scintimammography (SC), magnetic resonance imaging (MRI), and ultrasonography (US)) what are the sensitivity and specificity of the tests for diagnosis of breast cancer in women presenting with: a) An abnormal mammogram, overall and by BIRADS classification or other relevant clinical classification (e.g., presence or absence of calcification, well circumscribed lesions, etc.)b) A palpable breast abnormalityc) What percentage of women in the studies in this question were age 65 or older, and do sensitivity and specificity vary by older vs. younger than age 65?** | **Key Question 1 - What is the accuracy (expressed as sensitivity, specificity, predictive values, and likelihood ratios) of noninvasive tests for diagnosis of breast cancer in women referred for further evaluation after identification of a possible breast abnormality on routine screening (mammography and/or clinical or self-detection of a palpable lesion)? The noninvasive tests to be evaluated are: Ultrasound (conventional B-mode, color Doppler, power Doppler, tissue harmonics, and tomography)Magnetic resonance imaging (MRI) with breast-specific coils and gadolinium-based contrast agents, with or without computer-aided diagnosis (CADx) Positron emission tomography (PET) with 18-fluorodeoxyglucose (FDG) as the tracer, with or without concurrent computed tomography (CT) scans Scintimammography (SMM) with technetium-99m sestamibi (MIBI) as the tracer, including Breast Specific Gamma Imaging (BSGI)** |  |  |
| To place the tests’ accuracy information into perspective, an average woman in the U.S. who has an abnormal mammogram requiring a biopsy for evaluation has approximately a 20-percent risk of cancer. For women at this average level of risk of cancer after an abnormal mammogram, based upon the tests' negative likelihood ratios: * For every 1,000 women who had a negative PET scan, about 924 women would have avoided an unnecessary biopsy, but 76 women would have missed cancers. * For every 1,000 women who had a negative scintimammogram, about 907 women would have avoided an unnecessary biopsy, but 93 women would have missed cancers. (These numbers are for nonpalpable lesions only; numbers could not be calculated for all lesions.) * For every 1,000 women who had a negative MRI, about 962 women would have avoided an unnecessary biopsy, but 38 women would have missed cancers. * For every 1,000 women who had a negative US, about 950 women For every 1,000 women who had a negative US, about 950 women would have avoided an unnecessary biopsy, but 50 women would have missed cancers. | This CER is an update of a CER finalized in 2006.7 The updated results are, in general, very similar to the findings of the 2006 report. For MRI, in 2006 we found that the sensitivity was 92.5 percent and the specificity was 75.5 percent; the updated evidence base supported estimates of 91.7 percent sensitivity and 77.5 percent specificity. In both reports, MRI was found to be less sensitive (approximately 85%) for evaluation of microcalcifications than for evaluation of lesions in general. For PET, in 2006 we found that the sensitivity was 82.2 percent and the specificity was 78.3 percent; the updated evidence base supported estimates of 83.0 percent sensitivity and 74.0 percent specificity. In the updated report we attempted to evaluate the accuracy of PET/CT, but only one study that met the inclusion criteria was identified. For scintimammography, the updated evidence base identified a sensitivity of 84.7 percent, much higher than the sensitivity estimate from 2006 of 68.7 percent. Specificity was estimated at 84.8 percent in 2006, and at 77.0 percent in the update; however, the confidence intervals around the updated estimate of specificity are wide. It is possible that improvements in the technology in the last few years improved the sensitivity of the technique. For ultrasound, in 2006 we evaluated a relatively small set of studies of B-mode grayscale ultrasound, and estimated a sensitivity of 86.1 percent and a specificity of 66.4 percent. The update included a significantly expanded evidence base on B-mode grayscale ultrasound, and identified a sensitivity of 92.4 percent and specificity of 75.8 percent. In the update we included numerous other types of ultrasound, including power and color Doppler ultrasound, that were not studied in the 2006 report.  The probability that a woman actually has cancer (invasive or in situ) even after a finding of “benign” on MRI depends on her probability of having cancer before undergoing the test. Bayes’ theorem and the summary likelihood ratios indicate that if a woman with an estimated 5 to 10 percent chance of having cancer undergoes MRI and has a finding of “benign” she will then have an estimated 1 percent chance of having cancer; a woman with an estimated 20 percent chance of having cancer who has a finding of “benign” on MRI will then have an estimated 3 percent chance of having cancer; and a woman with an estimated 50 percent chance of having cancer who has a finding of “benign” on MRI will then have an estimated 10 percent chance of having cancer.  The probability that a woman actually does have cancer (invasive or in situ) even after a finding of “benign” on PET depends on her probability of having cancer before undergoing the test. Bayes’ theorem and the summary likelihood ratios indicate that if a woman with an estimated 5 percent chance of having cancer undergoes PET and has a finding of “benign” she will then have an estimated 1 percent chance of having cancer; a woman with an estimated 20 percent chance of having cancer who has a finding of “benign” on PET will then have an estimated 6 percent chance of having cancer; and a woman with an estimated 50 percent chance of having cancer who has a finding of “benign” on PET will then have an estimated 19 percent chance of having cancer.  The probability that a woman actually does have cancer (invasive or in situ) even after a finding of “benign” on scintimammography depends on her probability of having cancer before undergoing the test. Bayes’ theorem and the summary likelihood ratios indicate that if a woman with an estimated 5 percent chance of having cancer undergoes scintimammography and has a finding of “benign” she will then have an estimated 1 percent chance of having cancer; a woman with an estimated 20 percent chance of having cancer who has a finding of “benign” on scintimammography will then have an estimated 5 percent chance of having cancer; and a woman with an estimated 50 percent chance of having cancer who has a finding of “benign” on scintimammography will then have an estimated 17 percent chance of having cancer.  The probability that a woman actually does have cancer (invasive or in situ) even after a finding of “benign” on ultrasound depends on her probability of having cancer before undergoing the test. Bayes’ theorem and the summary likelihood ratios indicate that if a woman with an estimated 5 to 10 percent chance of having cancer undergoes B-mode grayscale ultrasound and has a finding of “benign” she will then have an estimated 1 percent chance of having cancer; a woman with an estimated 20 percent chance of having cancer who has a finding of “benign” on B-mode grayscale ultrasound will then have an estimated 2 percent chance of having cancer; and a woman with an estimated 50 percent chance of having cancer who has a finding of “benign” on B-mode | It is difficult to estimate whether this conclusion is still valid or not, since it consists of calculations made based on operating characteristics of the test with “average” level of cancer risk. Using data from the Peters, 2008 meta-analysis, the “missed cancers” number would be 20, not 38. Therefore, this conclusion is possibly out of date, although probably modestly so. | Good |
| Although all of the technologies evaluated could reduce the need for biopsy in women with an abnormal mammogram who do not have cancer, each would miss some cancers. | There was a great deal of heterogeneity (I2 = 93%) in the reported data. We were unable to identify with meta-regression any study- related characteristics that explained this heterogeneity, such as consecutive enrollment of patients, blinding of the diagnostic test reader to patient history/other clinical information, and use of the gold standard (biopsy) as the reference standard. | Conclusion is still valid and this portion of the CER does not need updating. | Good |
| **Key Question 2 - For women with relevant demographic risk factors (e.g., age, family history) and clinical risk factors (e.g., BIRADS status or morphologic characteristics of the lesion), what are the positive and negative predictive values of the above diagnostic tests?** | **Key Question 2 - Are there demographic (e.g., age) and clinical risk factors (e.g., morphologic characteristics of the lesion) that affect the accuracy of the tests considered in Key Question 1?** |  |  |
| In general, the higher a woman’s risk of cancer is before undergoing a noninvasive test, the higher is the risk that she has cancer even if the test is negative.  If a less than 2-percent risk of having breast cancer with a negative diagnostic test is considered an acceptable level of risk for a diagnostic test to reliably preclude biopsy, none of these tests was sufficiently accurate to replace biopsy for women at average risk of breast cancer. | Two studies evaluated only patients with palpable breast masses,57,62 one study evaluated only patients with non-palpable breast masses,63 and one study evaluated only patients with microcalcifications detected on x-ray mammography.61 With so few studies reporting on each category, evidence-based conclusions are difficult to support. None of the studies reported outcomes by patient demographics or any other clinical risk factors that may have affected the accuracy of SMM.  For all of the technologies evaluated in this assessment, only women with a low suspicion of malignancy after standard-of-care workup might be expected to experience a change in management decisions as a result of additional noninvasive imaging. A woman with a ≤12 percent suspicion of malignancy who has benign findings on MRI could have her suspicion of malignancy drop below the 2 percent threshold, and therefore she might be assigned to short-interval imaging followup management rather than tissue sampling management; a woman with a 1 percent suspicion of malignancy who has benign findings on MRI could have her suspicion of malignancy drop to near 0 percent and therefore she might be assigned to return to normal screening rather than short-interval followup imaging.  Therefore, if the 2 percent threshold is chosen, the use of noninvasive imaging in addition to standard workup may be clinically useful for diagnostic purposes only for women with a low suspicion of malignancy. When choosing which noninvasive imaging technology to use for this purpose, diagnostic B-mode grayscale ultrasound and MRI appear to be more accurate than PET, scintimammography, or the other types of ultrasound (e.g., Doppler) that were evaluated in this comparative effectiveness review. | Conclusion is probably out of date and this portion of the CER may need updating based on new data on MRI and US. | Good. The updated report does not use the same "average" categorization, but concludes that MRI and B-mode grayscale ultrasound are clinically useful in women with a low suspicion of malignancy. |
| **Key Question 3 - Are there other factors that affect the accuracy or acceptability of the tests considered in Questions 1 and 2?** | **Key Question 3 - Are there other factors and considerations (e.g., safety, care setting, patient preferences, ease of access to care) that may affect the accuracy or acceptability of the tests considered in Key Questions 1 and 2?** |  |  |
| Based on results for only nonpalpable lesions (usually detected by mammography), data were insufficient to estimate the accuracy of PET scanning, MRI, or US. Scintimammography was not sufficiently accurate to avoid biopsy in women at average risk as judged by the acceptability standard of less than a 2-percent risk of breast cancer with a negative diagnostic test. Based on results for only palpable lesions, data were insufficient to estimate the accuracy of PET scanning, MRI, ultrasound, and scintimammography. | **MRI.** One study reported the accuracy of MRI images interpreted with and without a Computer Aided Diagnosis (CAD) software system.12 The study reported virtually no difference in either sensitivity (77.4% vs. 78.9%) or specificity (73.2% vs. 73.2%) with or without CAD assistance. **Positron Emission Tomography.** None of the seven studies on stand-alone PET scanning or the one study on PET with CT reported information that addressed this question. **Scintimammography.** None were identified.  **Ultrasound.** None were identified. | Conclusion is possibly out of date and this portion of the CER may need updating based on the new meta-analysis. It would need to be reviewed to assess whether data can be stratified. | Fair. New data was scanned and did not materially effect the conclusions. |

| **Original Key Questions/Conclusions** | **Updated Key Questions/Conclusions (2011-2012)** | **2009 Prediction** | **Concordance** |
| --- | --- | --- | --- |
| **Comparative Effectiveness and Safety of Analgesics for Osteoarthritis (Original report date - Sep 2006 and Update report date - Oct 2011)** | | | |
| **Key Question 1 - What are the comparative benefits and harms of treating osteoarthritis 20 with oral medications or supplements? How do these benefits and harms change with dosage and duration of treatment, and what is the evidence that alternative dosage strategies, such as intermittent dosing and drug holidays, affect the benefits and harms of oral medication use? (Note: This question addresses the therapeutic benefits of long-term use for the condition osteoarthritis. However, the question does address all harms associated with NSAID use, including** | **Key Question 1 - What are the comparative benefits and harms of treating osteoarthritis with oral medications or supplements? How do these benefits and harms change with dosage and duration of treatment? The only benefits considered here are improvements in osteoarthritis symptoms. Evidence of harms associated with the use of NSAIDs includes studies of these drugs for treating osteoarthritis or rheumatoid arthritis and for cancer prevention. Oral agents include: COX-2 selective NSAIDs: o Celecoxib Partially selective NSAIDs: o Etodolac, Meloxicam, Nabumetone Non-aspirin, nonselective NSAIDs: Diclofenac, Diflunisal, Fenoprofen, Flurbiprofen, Ibuprofen, Indomethacin, Ketoprofen, Ketorolac, Meclofenamate sodium, Mefenamic acid, Naproxen, Oxaprozin, Piroxicam, Sulindaco, Tolmetin Aspirin and salsalate: Aspirin, Salsalate Acetaminophen and supplements: Acetaminophen, Chondroitin, Glucosamine** |  |  |
| There are no clear differences between various nonaspirin, nonselective NSAIDs or partially selective NSAIDs (meloxicam, nabumetone, etodolac) in efficacy for pain relief or improvement in function. | No clear difference in efficacy for pain relief, or withdrawals due to lack of efficacy. Meloxicam was associated with no clear difference in efficacy compared to nonselective NSAIDs in eleven head-to-head trials of patients with osteoarthritis, but a systematic review that included trials of patients with osteoarthritis or rheumatoid arthritis found lesser effects on pain compared to nonselective NSAIDs (difference 1.7 points on a 10 point VAS pain scale) and withdrawals due to lack of efficacy (RR 1.5,95% CI 1.2 to 1.7). Etodolac and nonselective NSAIDs were associated with no statistically significant differences on various efficacy outcomes in several systematic reviews of patients with osteoarthritis, with consistent results reported in 7 trials not included in the systematic reviews. Nabumetone was similar in efficacy to nonselective NSAIDs in two trials. No difference in efficacy between various non-aspirin, nonselective NSAIDs.No difference in efficacy between aspirin and salsalate in one head-to-head trial. No trial compared aspirin or salsalate vs. other NSAIDs. | Conclusion is still valid and this portion of the CER does not need updating. | Good |
| It is not clear whether celecoxib has fewer potential harms than nonselective NSAIDs when used longer than 3-6 months. | GI harms: Celecoxib was associated with a lower risk of ulcer complications (RR 0.23, 95% CI 0.07 to 0.76) and ulcer complications or symptomatic ulcers (RR 0.39, 95% CI 0.21-0.73) compared to nonselective NSAIDs in a systematic review of randomized trials. The systematic review included the pivotal, large, long-term CLASS study, in which celecoxib was superior to diclofenac or ibuprofen for ulcer complications or symptomatic ulcers at 6-month followup (2.1% vs. 3.5%, p=0.02), but not at 12-month followup. However, CLASS found difference in rates of ulcer complications alone at either 6 or 12 months. Other long-term followup data from randomized trials is lacking. A systematic review found celecoxib associated with a lower risk of upper GI bleeding or perforation compared to various nonselective NSAIDs based on 8 observational studies, though confidence interval estimates overlapped in some cases. CV harms: There was no increase in the rate of cardiovascular events with celecoxib vs. ibuprofen or diclofenac in CLASS (0.5% vs. 0.3%). In three systematic reviews of randomized trials, celecoxib was associated with increased risk of cardiovascular events compared to placebo (risk estimates ranged from 1.4 to 1.9).A systematic review of placebo-controlled trials with at least 3 years of planned followup found celecoxib associated with an increased risk of cardiovascular events (CV death, myocardial infarction, stroke, heart failure, or thromboembolic event) compared to placebo (OR 1.6, 95% CI 1.1 to 2.3). | Conclusion is probably out of date and this portion of the CER may need updating based on new data and expert opinion. | Good - updated CER reports that celcoxib is superior at 6 months to diclofenac or ibuprofen |
| Celecoxib is associated with an increased risk of myocardial infarction. Most of the CV events with celecoxib were reported in two large polyp-prevention trials. | About 3.7 additional cardiovascular events occurred for every 1,000 patients treated for one year with celecoxib instead of placebo, or 1 additional cardiovascular event for every 270 patients treated for 1 year with celecoxib instead of placebo. The risk was highest in patients prescribed celecoxib 400 mg twice daily compared to celecoxib 200 mg twice daily or 400 mg once daily. Much of the evidence for increased risks comes from two large colon polyp prevention trials. A network analysis of randomized trials and three large observational studies found celecoxib associated with no clear difference in risk of myocardial infarction compared to naproxen, ibuprofen, ordiclofenac; a fourth observational study found celecoxib associated with lower risk than ibuprofen or naproxen. 11 of13 large observational studies found celecoxib associated with no increased risk of myocardial infarction compared to nonuse of NSAIDs. An analysis of all serious adverse events in CLASS based on FDA data found no difference between celecoxib (12/100 patient-years), diclofenac (10/100 patient-years), and ibuprofen (11/100 patient-years). A retrospective cohort study found celecoxib and ibuprofen associated with neutral risk of hospitalization for acute myocardial infarction or GI bleeding compared to use of acetaminophen, but naproxen was associated with increased risk (HR 1.6, 95% CI 1.3 to 1.9.) | Conclusion is still valid and this portion of the CER does not need updating. | Good |
| Etoricoxib is associated with fewer GI adverse events (perforations, symptomatic ulcers, and bleeds) than nonselective NSAIDs. Reviews of RCTs suggest that etoricoxib has a similar CV safety profile compared to other NSAIDs, with the possible exception of naproxen. Definitive conclusions are not possible because of small numbers of CV events. | No comparable conclusion in the update as etoricoxib was not given FDA approval. | Conclusion is possibly out of date and this portion of the CER may need updating based on diversity of expert opinion. | Good - Etoricoxib is not included in the updated review, as it was rejected by FDA for approval. |
| Results from one large trial found fewer adverse GI events with lumiracoxib than with naproxen and ibuprofen. Too few events have been reported in RCTs to accurately assess CV risk associated with lumiracoxib. | No comparable conclusion since lumiracoxib was not included in the update. | Conclusion is out of date and this portion of the CER needs updating since lumiracoxib is not FDA approved and has been withdrawn from the market of several countries. | Good |
| Meloxicam - There were no significant differences in risks of serious GI events or CV risk. | GI harms: Meloxicam (primarily at a dose of 7.5 mg/day) was associated with a lower risk of ulcer complications or symptomatic ulcers compared to various nonselective NSAIDs in 6 trials included in a systematic review (RR 0.53,95% CI 0.29 to 0.97), but the difference in risk of ulcer complications alone did not reach statistical significance (RR0.56, 95% CI 0.27 to 1.2). | Conclusion is still valid and this portion of the CER does not need updating. | Good |
| Nabumetone or etodolac - There was insufficient evidence to make reliable judgments about relative GI safety and no evidence on CV safety. | Etodolac (primarily at a dose of 600 mg/day) was associated with a lower risk of ulcer complications or symptomatic ulcer compared to various nonselective NSAIDs in 9 trials included in a systematic review (RR 0.32, 95% CI 0.15 to 0.71), but the difference in risk of ulcer complications alone did not reach statistical significance (RR 0.39, 95% CI 0.12 to 1.2) and the number of events was very small. Evidence was insufficient to make reliable judgments about GI safety of nabumetone.CV harms: One observational study evaluated etodolac and nabumetone, but estimates were imprecise. | Conclusion is out of date and this portion of the CER needs updating to reflect change in labeling due to addition of FDA boxed warning label. | Good. The following box warning on etidolac. The box warning was: Etodolac 1/18/2006 - Revised label to add a boxed warning to address possible CV risks as well as known GI risks |
| No clear difference in GI safety was found among nonselective NSAIDs at commonly used doses. | GI harms: COX-2 selective NSAIDs as a class were associated w/ a similar reduction in risk of ulcer complications vs. naproxen (RR 0.34, 95% CI 0.24 to 0.48), ibuprofen (RR 0.46, 95% CI 0.30 to 0.71), and diclofenac (RR 0.31, 95% CI 0.06 to 1.6) in a syst. review of randomized trials. Evidence from randomized trials on comparative risk of serious GI harms associated with other nonselective NSAIDs is sparse. In large observational studies, naproxen was associated with a higher risk of serious GI harms than ibuprofen in 7 studies. Comparative data on GI harms with other nonselective NSAIDs was less consistent. | Conclusion is still valid and this portion of the CER does not need updating. | Good |
| The CV safety of naproxen is moderately superior to that of any COX-2 selective NSAID. The CV safety of nonselective NSAIDs other than naproxen (data primarily on ibuprofen and diclofenac) was similar to that of COX-2 selective NSAIDs. | CV harms: An indirect analysis of randomized trials found ibuprofen (RR 1.5, 95% CI 0.96 to 2.4) and diclofenac (RR 1.6, 95% CI 1.1 to 2.4), but not naproxen (RR 0.92, 95% CI 0.67 to 1.3) associated with an increased risk of myocardial infarction relative to placebo. 1 additional myocardial infarction occurred for about every 300 patients treated for 1 year with celecoxib instead of naproxen. A network analysis of randomized trials reported consistent results with regard to CV events (nonfatal myocardial infarction, nonfatal stroke, or cardiovascular death; ibuprofen: RR 2.3, 95% CI 1.1 to 4.9; diclofenac: RR 1.6, 95% CI 0.85 to 3.0 and naproxen: RR 1.2, 955 CI 0.78 to 1.9). An Alzheimer‘s disease prevention trial was stopped early due to a trend towards increased risk of myocardial infarction (HR 1.5, 95% CI 0.69 to 3.2) vs. placebo, but did not employ pre specified stopping protocols. In most large observational studies, naproxen was associated with a neutral effect on risk of serious CV events. | Conclusion is still valid and this portion of the CER does not need updating. | Good |
| Aspirin is associated with a lower risk of thromboembolic events and a higher risk of GI bleeds compared to placebo or nonuse when given in long-term prophylactic doses. There is insufficient evidence to assess the balance of GI and CV safety of higher dose aspirin as used for pain relief compared with nonaspirin NSAIDs. | GI harms: A systematic review of individual patient trial data found aspirin associated with increased risk of major GI and other extra cranial bleeding when given for primary prevention of vascular events (RR 1.5, 95% CI 1.3 to 1.8, absolute risk 0.10% vs. 0.07%). Observational studies showed a similar risk of upper GI bleeding with aspirin and non-aspirin, nonselective NSAIDs. CV harms: Aspirin reduced the risk of vascular events in a collaborative meta-analysis of individual patient data from18 randomized controlled trials (0.51% aspirin vs. 0.57% control per year, p=0.0001 for primary prevention and 6.7% vs. 8.2% per year, p<0.0001 for secondary prevention). | Conclusion is still valid and this portion of the CER does not need updating. | Good |
| Almost no data are available on CV safety for salsalate. | No randomized trial or observational study evaluated risk of serious GI or CV harms with salsalate. | Conclusion is still valid and this portion of the CER does not need updating. | Good |
| All NSAIDs and COX-2 inhibitors can cause or aggravate hypertension, congestive heart failure (CHF), edema, and impaired renal function | All NSAIDs are associated with deleterious effects on blood pressure, edema, and renal function. No clear evidence of clinically relevant, consistent differences between celecoxib, partially selective, and nonselective NSAIDs in risk of hypertension, heart failure, or impaired renal function. | Conclusion is possibly out of date and this portion of the CER may need updating based on upcoming publication of new evidence (article by Winkelmayer, 2008) | Fair - the article identified in the surveillance was: Winkelmayer WC, Waikar SS, Mogun H, Solomon DH. Nonselective and cyclooxygenase-2-selective NSAIDs and acute kidney injury. Am J Med. Dec 2008;121(12):1092-1098. However, the update report included observational studies of adverse events with sample size greater than 1,000 only if the adverse event was CV or GI, not renal |
| Among currently marketed NSAIDs, only diclofenac was associated with a significantly higher rate of liver-related discontinuations compared with placebo. | Several NSAIDs associated with high rates of hepatotoxicity have been removed from the market. A systematic review found clinically significant hepatotoxicity rare with currently available NSAIDs. A systematic review of randomized trials found no difference between celecoxib, diclofenac, ibuprofen, and naproxen in clinical hepatobiliary adverse events, though diclofenac was associated with the highest rate of hepaticlaboratory abnormalities (78/1,000 patient-years, vs. 16 to28/1,000 patient-years for the other NSAIDs). Another systematic review found diclofenac associated with the highest rate of aminotransferase elevations compared to placebo (3.6% vs. 0.29%, compared to <0.43% with other NSAIDs). | Conclusion is still valid and this portion of the CER does not need updating. | Good |
| Uncertainty remains regarding the comparative tolerability of salsalate and nonselective NSAIDs. | In a systematic review of randomized trials, the only relatively consistent finding regarding the tolerability of different nonselective NSAIDs was that indomethacin was associated with higher rates of toxicity than other NSAIDs (statistical significant unclear). | Conclusion is still valid and this portion of the CER does not need updating. | Good |
| Acetaminophen is modestly inferior to NSAIDs for pain and function. Compared with NSAIDs, acetaminophen had fewer GI side effects and serious GI complications. Acetaminophen may be associated with modest increases in blood pressure and renal dysfunction. | Acetaminophen is consistently modestly inferior to NSAIDs for reducing pain and improving function in randomized trials included in multiple systematic reviews. Acetaminophen is superior to NSAIDs for GI side effects (clinical trials data) and GI complications (observational studies). Some observational studies found acetaminophen associated with modest increases in blood pressure or higher risk of renal dysfunction compared to NSAIDs, but results may besusceptible to confounding by indication. One observational study found risk of acute myocardial infarction similar in users of acetaminophen compared to users of NSAIDs Acetaminophen may cause elevations of liver enzymes at therapeutic doses in healthy persons; comparative hepatic safety has not been evaluated. | Conclusion is still valid and this portion of the CER does not need updating. | Good |
| One good-quality, prospective observational study found an increased risk of CV events with heavy use of acetaminophen that was similar to the risk associated with heavy use of NSAIDs. | No randomized trial evaluated the association between acetaminophen use and myocardial infarction or other thromboembolic CV events. An analysis from the large, prospective Nurses’ Health Study found heavy use of acetaminophen (more than 22 days/month) associated with an increased risk of CV events (RR 1.4, 95% CI 1.1 to 1.6) similar to that with heavy use of NSAIDs (RR 1.4, 95% CI 1.3 to 1.6). Dose- and frequency-dependent effects were both significant. A new retrospective cohort study found no difference in risk of acute myocardial infarction between celecoxib, ibuprofen, diclofenac, or naproxen versus acetaminophen. | Conclusion is still valid and this portion of the CER does not need updating. | Good |
| Glucosamine and chondroitin were generally well tolerated and no serious adverse events were reported in clinical trials. | Seven randomized trials showed no clear difference between glucosamine vs. oral NSAIDs for pain or function. One randomized trial showed no difference between chondroitin vs. an oral NSAID.A systematic review including recent, higher-quality trials found glucosamine associated with statistically significant but clinically insignificant beneficial effects on pain (-0.4 cm on a10 cm scale, 95% CI -0.7 to -0.1) and joint space narrowing(-0.2 mm, 95% CI -0.3 to 0.0) compared to placebo. The systematic review reported similar results for chondroitin. A recent large, good-quality NIH-funded trial found the combination of pharmaceutical grade glucosamine hydrochloride and chondroitin sulfate modestly superior to placebo only in an analysis of a small subset of patients with at least moderate baseline pain. Older trials showed a greater benefit with glucosamine or chondroitin, but were characterized by lower quality. For glucosamine, the best results have been reported in trials sponsored by the manufacturer of a European, pharmaceutical grade product (no pharmaceutical grade glucosamine available in the United States). | Conclusion is still valid and this portion of the CER does not need updating. | Good |
| We found no studies evaluating the GI or CV safety of alternative dosing strategies (such as alternate day dosing, once daily versus twice daily dosing, or periodic drug holidays).The risk of GI bleeding increases with higher doses of nonselective NSAIDs. | One small trial found continuous celecoxib slightly more effective than intermittent use on pain and function, and similar rates of withdrawals due to adverse events. No trial was designed to assess serious GI or CV harms associated with intermittent dosing strategies. | Conclusion is possibly out of date and this portion of the CER may need updating based on new data. | Good - there is now the one celecoxib alternative dosing study, so the conclusion that there were no studies was out of date |
| Higher doses of celecoxib were associated with increased CV risk, but could not determine the effects of dose on CV risk associated with rofecoxib due to low numbers of events at lower doses. Most trials of nonselective NSAIDs involved high doses. | Higher doses of NSAIDs were associated with greater efficacy for some measures of pain relief, and in some trials with greater withdrawals due to adverse events A meta-analysis of 41 randomized trials found no clear association between longer duration of therapy with COX-2selective NSAIDs and increase in the relative risk of CV events. The meta-analysis found higher doses of celecoxib associated with increased risk of cardiovascular events, but most events occurred in the long-term polyp prevention trials. Almost all of the cardiovascular events in trials of celecoxib were reported in long-term trials of colon polyp prevention. Large observational studies showed no association between higher dose and longer duration of nonselective NSAID therapy and increased risk of cardiovascular events. Many observational studies found that risk of GI bleeding increased with higher doses of nonselective NSAIDs, but no clear association with duration of therapy. One small trial found continuous celecoxib slightly more effective than intermittent use on pain and function, and similar rates of withdrawals due to adverse events. No trial was designed to assess serious GI or CV harms associated with intermittent dosing strategies. | Conclusion is still valid and this portion of the CER does not need updating. | Good |
| **Key Question 2 - Do the comparative benefits and harms of oral treatments for osteoarthritis vary for certain demographic and clinical subgroups of patients?* Demographic subgroups include age, sex, and race.* Coexisting diseases include hypertension, edema, ischemic heart disease, heart failure; peptic ulcer disease; history of previous bleeding due to NSAIDs.* Concomitant medication use includes anticoagulants.** | **Key Question 2 - Do the comparative benefits and harms of oral treatments for osteoarthritis vary for certain demographic and clinical subgroups of patients?-Demographic subgroups: age, sex, and race-Coexisting diseases: CV conditions, such as hypertension, edema, ischemic heart disease, heart failure; peptic ulcer disease; history of previous gastrointestinal bleeding (any cause); renal disease; hepatic disease; diabetes; obesity-Concomitant medication use: antithrombotics, corticosteroids, antihypertensives, selective serotonin reuptake inhibitors (SSRI)** |  |  |
| GI and CV complication rates are higher among older patients and those with predisposing comorbid conditions, but there is no evidence that the relative safety of different NSAIDs varies according to baseline risk. Compared to nonuse of NSAIDs, one additional death per 1 year of use occurred for every 13 patients treated with rofecoxib, 14 with celecoxib, 45 with ibuprofen, and 24 with diclofenac in one large, population-based observational study of high-risk patients with acute myocardial infarction. There is no evidence that the comparative safety or efficacy of specific selective or nonselective NSAIDs varies depending on age, gender, or racial group, although data are sparse. | The absolute risks of serious GI and CV complications increase with age. Large observational studies that stratified patients by age found no clear evidence of different risk estimates for different age groups. However, because the event rates increases in older patients, even if the relative risk estimates are the same, the absolute event rates are higher. There is insufficient evidence on the comparative benefits and harms of different selective and nonselective NSAIDs in men compared to women, or in different racial groups. | Conclusion is still valid and this portion of the CER does not need updating. | Good |
| Among patients who had a recent episode of upper GI bleeding, there is good evidence that rates of recurrent ulcer bleeding are high (around 5 percent after 6 months) in patients prescribed celecoxib or a nonselective NSAID plus a PPI. | The risk of GI bleeding is higher in patients with prior bleeding. Two trials found high rates of recurrent ulcer bleeding in patients randomized to either celecoxib (4.9% to8.9% with 200 mg twice daily) or a nonselective NSAID + PPI(6.3%). | Conclusion is still valid and this portion of the CER does not need updating. | Good |
| Concomitant use of anticoagulants (e.g., warfarin) and any nonselective NSAID increases the risk of GI bleeding three- to six fold compared to anticoagulants alone. Reliable conclusions about the safety of selective NSAIDs used with anticoagulants are not possible due to flaws in existing observational studies, although there are case reports of serious bleeding events, primarily in the elderly. | Concomitant use of anticoagulants and nonselective NSAIDs increases the risk of GI bleeding three- to six fold compared with anticoagulant use without NSAIDs. The risk with concomitant celecoxib is not clear due to conflicting findings among observational studies, but may be increased in older patients. Reliable conclusions about the comparative safety of nonselective, partially selective, and COX-2 selective NSAIDs with concomitant anticoagulants could not be drawn due to small numbers of studies with methodological shortcomings. | Conclusion is still valid and this portion of the CER does not need updating. | Good |
| There was no difference in rates of ulcer complications between celecoxib and nonselective NSAIDs in the subgroup of patients who took aspirin. Concomitant low-dose aspirin use increased the rate of endoscopic ulcers in both patients on celecoxib and those on nonselective NSAIDs. Rofecoxib plus low-dose aspirin or ibuprofen alone were associated with similar risks of endoscopic ulcers which were significantly higher than those for placebo (6 percent) or aspirin alone. Compared to nonuse of aspirin, concomitant aspirin use did not ameliorate the increased risk of vascular events associated with COX-2 selective NSAIDs. | Concomitant use of aspirin appears to attenuate or eliminate the GI benefits of selective NSAIDs, resulting in risks similar to nonselective NSAIDs. Concomitant low-dose aspirin increased the rate of endoscopic ulcers by about 6 percent inpatients on celecoxib and those on nonselective NSAIDs in one meta-analysis. Evidence regarding the effects of concomitant aspirin use on CV risk associated with selective or nonselective NSAIDs is limited, though three polyp prevention trials of COX-2selective NSAIDS found that concomitant aspirin use did not attenuate the observed increased risk of CV events. Observational studies did not find increased CV risk with the addition of nonselective NSAIDs as a class to low-dose aspirin. Limited evidence suggests an increased risk of mortality with aspirin and concomitant ibuprofen compared to aspirin alone among high risk patients (HR 1.9, 95% CI 1.3 to2.9), but studies on effects of ibuprofen added to aspirin on MI risk in average risk patients were inconsistent and did not clearly demonstrate increased risk. | Conclusion is possibly out of date and this portion of the CER may need updating based on expert opinion. | Fair. The studies suggested by the experts ended up being either ineligible for inclusion in the update or were already included in the original CER. |
| **Key Question 3 - What is the evidence that the gastrointestinal harms of NSAID use are reduced by co-prescribing of H2-antagonists, misoprostol, or proton pump inhibitors?** | **Key Question 3 - What are the comparative effects of coprescribing H2 receptor antagonists, misoprostol, or proton pump inhibitors on the gastrointestinal harms associated with NSAID use?** |  |  |
| Consistent evidence found coprescribing of PPIs to be associated with the lowest rates of endoscopically detected duodenal ulcers relative to gastroprotective agents. Coprescribing of misoprostol is associated with similar rates of endoscopically detected gastric ulcers as coprescribing of PPIs. While misoprostol offers the advantage of being the only gastroprotective agent to reduce rates of perforation, obstruction, or bleeding, there is a high rate of withdrawals due to adverse GI symptoms. | Misoprostol was the only gastroprotective agent to reduce risk of ulcer complications compared to placebo in patients with average risk of GI bleeding prescribed nonselective NSAIDs, but was also associated with a higher rate of withdrawals due to adverse GI symptoms. Coprescribing of PPIs, misoprostol, and H2-antagonists all reduced the risk of endoscopically detected gastric and duodenal ulcers compared to placebo in patients prescribed a nonselective NSAID. | Conclusion is still valid and this portion of the CER does not need updating. | Good |
| The risk of endoscopic duodenal ulcers for standard-dose H2 blockers was lower than placebo, similar to misoprostol, and higher than omeprazole. Standard dosages of H2 blockers were associated with no reduction of risk for gastric ulcers relative to placebo. Double (full) dose H2 blockers were associated with a lower risk of endoscopic gastric and duodenal ulcers relative to placebo. It is unknown how full-dose H2 blockers compare to other antiulcer medications. | In direct comparisons, coprescribing of PPIs in patients with increased risk of GI bleeding who were prescribed a nonselective NSAID was associated with a lower risk of endoscopically detected duodenal ulcers compared tomisoprostol or H2-antagonists, a lower risk of endoscopically detected gastric ulcers compared to H2-antagonists, and a similar risk of endoscopically detected gastric ulcers compared to misoprostol. Coprescribing of misoprostol was associated with a lower risk of endoscopically detected gastric ulcers compared to ranitidine, and a similar reduction in risk of endoscopically detected duodenal ulcers. Compared to placebo, double (full) dose H2-antagonists maybe more effective than standard dose for reducing endoscopically detected gastric and duodenal ulcers. Celecoxib alone was associated with fewer decreases in hemoglobin (> 2 g/dl) without overt GI bleeding compared with diclofenac plus a PPI. Celecoxib plus a PPI may reduce the risk of endoscopic ulcers and ulcer complications compared to celecoxib alone in average risk persons. | Conclusion is still valid and this portion of the CER does not need updating. | Good |
| **Key Question 4 - What are the comparative benefits and harms of treating osteoarthritis with oral medications as compared with topical preparations? Topical preparations include: capsaicin, diclofenac, ibuprofen, ketoprofen, and salicylate.** | **Key Question 4 - What are the comparative benefits and harms of treating osteoarthritis with oral medications compared with topical preparations, or of different topical medications compared with one another?** |  |  |
| Topical NSAIDs were similar to oral NSAIDs for pain relief in trials primarily of patients with osteoarthritis of the knee, with topical diclofenac (often with dimethyl sulphoxide [DMSO], a drug not approved for use in humans in the United States). Topical ibuprofen was superior to placebo in several trials. Consistent evidence from good-quality trials, systematic reviews, and observational studies found topical NSAIDs to be associated with increased local adverse events compared with oral NSAIDs. Total adverse events and withdrawal due to adverse events were similar. Data from one good-quality trial found topical NSAIDs superior to oral NSAIDs for GI events, including severe events, and changes in hemoglobin. Topical salicylates were no better than placebo in higher quality placebo-controlled trials. Compared to placebo, one additional patient achieved pain relief for every eight that used topical capsaicin in a good-quality meta-analysis, but capsaicin was associated with increased local adverse events and withdrawals due to adverse events. | Three head-to-head trials found topical diclofenac similar to oral NSAIDs for efficacy in patients with localized osteoarthritis. Topical NSAIDs were associated with a lower risk of GI adverse events and higher risk of dermatologic adverse compared to oral NSAIDs. There was insufficient evidence to evaluate comparative risks of GI bleeding or CV events. Other topical NSAIDs evaluated in head-to-head trials have not been FDA-approved. No head-to-head trials compared topical salicylates or capsaicin to oral NSAIDs for osteoarthritis. Topical salicylates were no better than placebo in two trials of patients with osteoarthritis included in a systematic review, and associated with increased risk of local adverse events when used for any acute or chronic pain condition. Topical capsaicin was superior to placebo (NNT 8.1), but associated with increased local adverse events and withdrawals due to adverse events (13% vs. 3%, RR 4.0, 95% CI 2.3 to 6.8). | Conclusion is still valid and this portion of the CER does not need updating. | Good |

| **Original Key Questions/Conclusions** | **Updated Key Questions/Conclusions (2011-2012)** | **2009 Prediction** | **Concordance** |
| --- | --- | --- | --- |
| **Efficacy and Comparative Effectiveness of Off-Label Use of Atypical Antipsychotics (Original report date - Jan 2007 and Update report date - Sep 2011)** | | | |
| **Key Question 1 - What are the leading off-label uses of atypical antipsychotics in the literature?** | **Key Question 1 - What are the leading off-label uses of atypical antipsychotics in utilization studies? How have trends in utilization changed in recent years, including inpatient versus outpatient use? What new uses are being studied in trials?** |  |  |
| The most common off-label uses of atypical antipsychotics found in the literature were treatment of depression, obsessive-compulsive disorder, posttraumatic stress disorder, personality disorders, Tourette's syndrome, autism, and agitation in dementia. In October 2006, risperidone was approved for use in autism. | Atypicals have been studied as off-label treatment for the following conditions: ADHD, anxiety, dementia in elderly patients, depression, eating disorders, insomnia, OCD, personality disorder, PTSD, substance use disorders, and Tourette’s syndrome. Off-label use of atypical antipsychotics in various settings has increased rapidly since their introduction in the 1990s; risperidone, quetiapine, and olanzapine are the most common atypicals prescribed for off-label use. One recent study indicated that the 2005 regulatory warning from the FDA and Health Canada was associated with decreases in the overall use of atypical antipsychotics, especially among elderly dementia patients. Use of atypicals in the elderly is much higher in long-term care settings than in the community. Atypicals are frequently prescribed to treat PTSD in the U.S. Department of Veterans Affairs health system. At least 90 percent of antipsychotics prescribed to children are atypical, rather than conventional antipsychotics. The majority of use is off-label. No off-label use of the newly approved atypicals (asenapine, iloperidone, and paliperidone) was reported in the utilization literature. | Conclusion is still valid, but AHRQ may wish to expand scope to anorexia depending on sponsor and public interest. | Good. Anorexia (eating disorders) was added to the update. Other off-label conditions were added as well, so the identification of one new off-label indication was a marker for more. |
| **Key Question 2 - What does the evidence show regarding the effectiveness of atypical antipsychotics for off-label indications, such as depression? How do atypical antipsychotic medications compare with other drugs for treating off-label indications?** | **Key Question 2 - What does the evidence show regarding the efficacy and comparative effectiveness of atypical antipsychotics for off-label indications? Sub-Key Question 2: How do atypical antipsychotic medications compare with other drugs, including first generation antipsychotics, for treating off-label indications?** |  |  |
| There is a small but statistically significant benefit for risperidone and aripiprazole on agitation and psychosis outcomes in dementia patients. The clinical benefits must be balanced against side effects and potential harms. | It is important to note that no trials of the three most recently FDA-approved atypicals (asenapine, iloperidone, and paliperidone) were found for off-label use. Aripiprazole, olanzapine, and risperidone have efficacy as treatment for behavioral symptoms of dementia. | Conclusion is possibly out of date and this portion of the CER may need updating. Although no experts felt the conclusion was out of date, we found new studies that reported olanzapine and quetiapine effective and two new meta-analyses. | Good. Olanzapine was added to the list of drugs with efficacy for dementia. |
| For SRI-resistant patients with major depressive disorder, combination therapy with an atypical antipsychotic plus an SRI antidepressant is not more effective than an SRI alone at 8 weeks. | Aripiprazole, quetiapine, and risperidone have efficacy as augmentation to SSRIs/SNRIs for major depressive disorder. Olanzapine and ziprasidone may also have efficacy. | Conclusion is probably out of date and this portion of the CER may need updating based on the new FDA approval, plus new literature and expert opinion. | Good. Studies now show efficacy for this indication. |
| Olanzapine alone was no better than placebo in improving symptoms at 6 or 12 weeks. Outcomes were too heterogeneous to allow pooling. | Olanzapine does not have efficacy as monotherapy for major depressive disorder. Quetiapine has efficacy as monotherapy for major depressive disorder. | Conclusion is still valid and this portion of the CER does not need updating. | Fair. The olanzapine conclusion is the same. The quetiapine conclusion is new. |
| In patients with major depressive disorder with psychotic features, olanzapine and olanzapine plus fluoxetine were compared with placebo for 8 weeks in 2 trials. There was a benefit for olanzapine alone. | There is no analogous conclusions because major depression with psychotic features was dropped from the update because it became an on-label indication | Conclusion is still valid and this portion of the CER does not need updating. | Not applicable for this update report, because the indication became on-label |
| For bipolar depression, olanzapine and quetiapine were superior to placebo in one study for each drug, but data are conflicting in two other studies that compared atypical antipsychotics to conventional treatment. The three olanzapine studies also assessed its efficacy as monotherapy. | There is no analogous conclusion because bipolar depression became an on-label indication. | Conclusion is still valid and this portion of the CER does not need updating. | Not applicable for this update report, because the indication became on-label. |
| We identified 12 trials of risperidone, olanzapine, and quetiapine used as augmentation therapy in patients w/ OCD who were resistant to standard treatment (nine trials were sufficiently similar clinically to pool). Atypical antipsychotics have a clinically important benefit (measured by the Yale-Brown Obsessive-Compulsive Scale) when used as augmentation therapy for OCD patients who fail to adequately respond to SRI therapy. There were too few studies of olanzapine augmentation to permit separate pooling for this drug. | Risperidone has efficacy in improving OCD symptoms when used as an adjunct to SSRI in treatment refractory patients. Olanzapine may also have efficacy. Quetiapine is more efficacious than ziprasidone and clomipramine for this purpose. Quetiapine and risperidone may be efficacious as augmentation to citalopram in OCD patients. | Conclusion is possibly out of date and this portion of the CER may need updating due to publication of aripiprazole trial. The results from the two new head to head trials could be added to the pooling performed in the original CER. | Good. These additional studies were added to the updated report. |
| We found four trials of risperidone and two trials of olanzapine of at least 6 weeks duration in patients with PTSD. There were three trials enrolling men with combat-related PTSD; these showed a benefit in sleep quality, depression, anxiety, and overall symptoms when risperidone or olanzapine was used to augment therapy with antidepressants or other psychotropic medication. There were three trials of olanzapine or risperidone as monotherapy for women with PTSD; the evidence was inconclusive regarding efficacy. | Risperidone is efficacious in reducing combat-related PTSD symptoms when used as an adjunct to primary medication. | Conclusion is still valid and this portion of the CER does not need updating. | Good |
| We identified five trials of atypical antipsychotic medications as treatment for borderline personality disorder & one trial as treatment for schizotypal personality disorder. Three RCTs each w/ no more than 60 subjects provide evidence that olanzapine is more effective than placebo & may be more effective than fluoxetine in treating borderline personality disorder. The benefit of adding olanzapine to dialectical therapy for borderline personality disorder was small. | Olanzapine had mixed results in 7 trials, aripiprazole was found efficacious in two trials, quetiapine was found efficacious in one trial, and ziprasidone was found not efficacious in one trial. | Conclusion is still valid and this portion of the CER does not need updating. | Good |
| Risperidone was more effective than placebo for the treatment of schizotypal personality disorder in one small trial. | Risperidone had mixed results when used to treat schizotypal personality disorder in two small trials. | Conclusion is still valid and this portion of the CER does not need updating. | Good |
| Aripiprazole was more effective than placebo for the treatment of borderline personality in one small trial. | Two studies have reported improvement in borderline personality disorders with aripirazole treatment. | Conclusion is still valid and this portion of the CER does not need updating. | Good |
| We found four trials of risperidone and one of ziprasidone for treatment of Tourette's syndrome. Risperidone was more effective than placebo in one small trial, and it was at least as effective as pimozide or clonidine for 8 to 12 weeks of therapy in the three remaining trials. The one available study of ziprasidone showed variable effectiveness compared to placebo. | Same as 2006: Risperidone is at least as efficacious as pimozide or clonidine for Tourette’s syndrome. | Conclusion is still valid and this portion of the CER does not need updating. | Good |
| Two trials support the superiority of risperidone over placebo in improving serious behavioral problems in children with autism. | No analogous conclusion, as autism was deleted as a target condition from the update report | Conclusion is still valid and this portion of the CER does not need updating. | Not applicable for this update since the condition was deleted |
|  | Quetiapine has efficacy as treatment for Generalized Anxiety Disorder. Risperidone may be efficacious in treating children with ADHD with no serious co-occurring disorders. Risperidone may be superior to methylphenidate in treating ADHD symptoms in mentally retarded children. Aripiprazole is inefficacious in reducing ADHD symptoms in children with bipolar disorder. Olanzapine and quetiapine have no efficacy in increasing body mass in eating disorder patients. Quetiapine may be inefficacious in treating insomnia. Aripiprazole is inefficacious in treating alcohol abuse/dependence. Quetiapine may also be inefficacious. Olanzapine is inefficacious in treating cocaine abuse/dependence. Risperidone may also be inefficacious. Aripiprazole is inefficacious in treating methamphetamine abuse/dependence. Risperidone is an inefficacious adjunct to methadone maintenance |  | New off label conditions, but for none did AAP have substantial clinical benefit |
| **Key Question 3 - What subset of the population would potentially benefit from off-label uses?** | **Key Question 3 - What subset of the population would potentially benefit from off-label uses? Do effectiveness and harms differ by race/ethnicity, gender, and age group? By severity of condition and clinical subtype?** |  |  |
| Other than specific populations listed in the finding for Key Question 2, there was insufficient information to answer this question. Therefore, it is included as a topic for future research. | There are insufficient data regarding efficacy, effectiveness, and harms to determine what subset of the population would potentially benefit from off-label uses of atypicals. Only one study conducted a subgroup analysis by gender; there were no studies that stratified by racial or ethnic group. Although many studies specified age in their inclusion criteria, few studies stratified results by age. Examination of the literature for differing efficacy of atypicals by clinical subsets did not reveal studies reporting subgroup analyses. Our own meta-analysis found efficacy for combat-related PTSD in men but not for PTSD in civilian women, although these data come from separate literatures, and head-to-head comparison of gender effects within study have not been performed. Due to the varying measures utilized in determining severity of illness, it was not possible to analyze treatment effects by severity of illness across any other condition. | Conclusion is still valid and this portion of the CER does not need updating. | Good |
| **Key Question 4 - What are the potential adverse effects and/or complications involved with off-label prescribing of atypical antipsychotics?** | **Key Question 4 - What are the potential adverse effects and/or complications involved with off-label prescribing of atypical antipsychotics? How do they compare within the class and with other drugs used for the conditions?** |  |  |
| Olanzapine patients are more likely to report weight gain than those taking placebo, other atypical antipsychotics, or conventional antipsychotics. | Weight gain elderly - More common in patients taking olanzapine and risperidone than placebo according to our metaanalysis. Weight gain in adults - More common in patients taking aripiprazole, olanzapine, quetiapine, and risperidone than placebo according to our meta-analysis. | Conclusion is still valid and this portion of the CER does not need updating. | Good |
| In a recently published meta-analysis death occurred in 3.5 percent of dementia patients randomized to receive atypical antipsychotics vs. 2.3 percent of patients randomized to receive placebo. The difference in risk for death was small but statistically significant. Sensitivity analyses did not show evidence for differential risks for individual atypical antipsychotics. In another recently published meta-analysis of six trials of olanzapine in dementia patients, differences in mortality between olanzapine and risperidone were not statistically significant, nor were differences between olanzapine and conventional antipsychotics. | Mortality in elderly - The difference in risk for death was small but statistically significant for atypicals, according to a 2006 metaanalysis which remains the best available estimate. Sensitivity analyses found no difference between drugs in the class. Patients taking atypicals had higher odds of mortality than those taking no antipsychotics in the two cohort studies that made that comparison. There are no trials or large observational studies of ziprasidone in this population; therefore, we cannot make conclusions regarding safety here. | While the conclusion is still valid, the strength of evidence supporting the conclusion has increased and therefore this may possibly need updating. | Good |
| In our pooled analysis of three RCTs of elderly patients with dementia, risperidone was associated with increased odds of cerebrovascular accident compared to placebo. This risk was equivalent to 1 additional stroke for every 31 patients treated in this patient population (i.e., number needed to harm of 31). The manufacturers of risperidone pooled four RCTs and found that cerebrovascular adverse events were twice as common in dementia patients treated with risperidone as in the placebo patients. In a separate industry-sponsored analysis of five RCTs of olanzapine in elderly dementia patients, the incidence of cerebrovascular adverse events was three times higher in olanzapine patients than in placebo patients. | More common in risperidone patients than placebo according to four PCTs pooled by the manufacturer. In our new meta-analysis of PCTs, risperidone was the only drug associated with an increase. More common in olanzapine than placebo according to five PCTs pooled by the manufacturer. | Conclusion is still valid and this portion of the CER does not need updating. | Good |
| We pooled three aripiprazole trials and four risperidone trials that reported extrapyramidal side effects (EPS) in elderly dementia patients. Both drugs were associated with an increase in EPS. | Head to head comparisons, more common in patients taking aripiprazole and risperidone patients than patients taking quetiapine in one large trial (CATIE-AD). For active comparisons, no evidence reported. For placebo comparisons, more common in patients taking risperidone, according to our metaanalysis. Quetiapine and aripiprazole were not associated with an increase. More common in olanzapine in one PCT. ziprasidone than placebo according to our meta-analysis. | Conclusion is still valid and this portion of the CER does not need updating. | Good. Any changes in estimates of harms were minor |
| Ziprasidone was associated with an increase in EPS when compared to placebo in a pooled analysis of adults with depression, PTSD, or personality disorders. | Head to head comparisons, no evidence reported. For active comparisons, less likely in patients taking quetiapine than mood stabilizers in one small trial. For active comparisons, less likely in patients taking olanzapine or aripiprazole than patients taking conventional antipsychotics in one trial each. For placebo comparisons, More common in patients taking aripiprazole, quetiapine, and ziprasidone than placebo according to our meta-analysis. | Conclusion is still valid and this portion of the CER does not need updating. | Good. Any changes in estimates of harms were minor |
| Risperidone was associated with increased weight gain compared to placebo in our pooled analyses of three trials in children/adolescents. Odds were also higher for gastrointestinal problems, increased salivation, fatigue, EPS, and sedation among these young risperidone patients. | Weight gain children - No head-to-head studies. For active comparisons, no difference between clonidine and risperidone in one trial. More common in patients taking risperidone in two PCTs. No difference in one small PCT of ziprasidone. | Conclusion is still valid and this portion of the CER does not need updating. | Good. Any changes in estimates of harms were minor |
| Compared to placebo, all atypicals were associated with sedation in multiple pooled analyses for all psychiatric conditions studied. | Sedation in elderly - More common in patients taking aripiprazole, olanzapine, quetiapine, and risperidone than placebo according to our meta-analysis. Sedation in children - Less common in aripiprazole patients than placebo patients in one PCT. No difference from placebo in one small PCT of ziprasidone. Sedation in adults - More common in patients taking aripiprazole, olanzapine, quetiapine, risperidone, and ziprasidone than placebo in our meta-analysis. | Conclusion is still valid and this portion of the CER does not need updating. | Good. Any changes in estimates of harms were minor |
| **Key Question 5 - What are the appropriate dose and time limit for off-label indications?** | **Key Question 5 - What is the effective dose and time limit for off-label indications?** |  |  |
| There was insufficient information to answer this question. Therefore, it is included as a topic for future research | There are too few studies comparing doses of atypical antipsychotic medications to draw a conclusion about a minimum dose needed. Most trials used flexible dosing, resulting in patients taking a wide range of doses. According to a meta-analysis we were able to conduct using the percentage of remitters and responders according to the MADRS as outcome, 150 mg quetiapine daily augmentation has equal efficacy as augmentation with 300 mg for patients with MDD who respond inadequately to SSRIs. More trials examining different doses of other atypicals for MDD would help guide clinicians in treating this population. In addition, more dosage trials for treating conditions such as OCD, PTSD, and anxiety disorder would allow for pooling and comparison of results. Though there is some trial data regarding duration of treatment in PTSD, eating disorders, and borderline personality disorder, the outcome of treatment appears to be the same regardless of reported followup time. | Conclusion is still valid and this portion of CER does not need updating. | Good |

| **Original Key Questions/Conclusions** | **Updated Key Questions/Conclusions (2011-2012)** | | **2009 Prediction** | **Concordance** |
| --- | --- | --- | --- | --- |
| **Comparative Effectiveness of Second-Generation Antidepressants in the Pharmacologic Treatment of Adult Depression (Original report date - Jan 2007 and Update report date - Dec 2011)** | | | | |
| **Key Question 1a. - For adults with major depressive disorder (MDD), dysthymia, or subsyndromal depressive disorders, do commonly used medications for depression differ in efficacy or effectiveness in treating depressive symptoms?** | | **Key Question 1a. - For adults with major depressive disorder (MDD), dysthymia, or subsyndromal depressive disorders, do commonly used medications for depression differ in efficacy or effectiveness in treating depressive symptoms?** |  |  |
| The relative risk (RR) of response was significantly greater for escitalopram than for citalopram. | | Citalopram versus escitalopram (5 published studies; 1,802 patients): For patients on escitalopram the odds ratio (OR) of response was statistically significantly higher than for patients on citalopram (OR, 1.47; 95% confidence interval [CI], 1.07 to 2.01). The number needed to treat (NNT) to gain 1 additional responder at week 8 with escitalopram compared with citalopram was 13 (95% CI, 8 to 39). These results are based on metaanalyses of head-to-head trials. Results of mixed-treatment comparisons, taking the entire evidence base on second-generation antidepressants into consideration, did not confirm these findings (OR, 0.51; 95% credible interval, 0.13 to 4.14). | Conclusion is still valid and this portion of the CER does not need updating. | Good |
| Fluoxetine vs. paroxetine: We did not find any statistically significant differences in effect sizes on the Hamilton Rating Scale for Depression (HAM-D) or response rates between fluoxetine and paroxetine. Paroxetine led to a higher rate of responders than fluoxetine. | | Fluoxetine versus paroxetine (5 studies; 690 patients): We did not find any statistically significant differences in response rates (OR, 1.08; 95% CI, 0.79 to 1.47) between fluoxetine and paroxetine. | Conclusion is still valid and this portion of the CER does not need updating. | Good |
| Fluoxetine vs. sertraline: Patients on sertraline had an additional, statistically nonsignificant treatment effect of a 0.75-point reduction (95-percent CI, –0.45-1.95) on the HAM-D scale compared with patients on fluoxetine. The relative risk of response was significantly greater for sertraline than for fluoxetine. | | Fluoxetine versus sertraline (4 studies; 940 patients): The odds ratio of response was statistically significantly higher for sertraline than for fluoxetine (OR, 1.42; 95% CI, 1.08 to 1.85). The NNT to gain 1 additional responder at 6 to 12 weeks with sertraline was 13 (95% CI, 8 to 58). | Conclusion is still valid and this portion of the CER does not need updating. | Good |
| Fluoxetine vs. venlafaxine: Patients on venlafaxine had an additional, statistically nonsignificant treatment effect of a 1.31-point reduction on the HAM-D scale compared with patients on fluoxetine. The relative risk of response was significantly greater for venlafaxine than for fluoxetine. | | Fluoxetine versus venlafaxine (6 studies; 1,197 patients): The odds ratio of response was statistically significantly higher for patients on venlafaxine than on fluoxetine (OR, 1.47; 95% CI, 1.16 to 1.86). | Conclusion is possibly out of date and this portion of the CER may need updating. Although we found only one new conflicting RCT, methods and inclusion criteria of new meta-analysis (Nemeroff, 2008) should be reviewed. | Fair The Nemeroff meta-analysis was reviewed but rejected for not assessing an outcome of interest. |
| Findings from indirect comparisons yielded no statistically significant differences in response rates. The precision of some of these estimates was low, leading to inconclusive results with wide confidence intervals. | | Results from direct and indirect comparisons based on 61 head-to head trials and 31 placebo-controlled trials indicate that no substantial differences in efficacy exist among second-generation antidepressants. Direct evidence from three effectiveness trials (one good) and indirect evidence from efficacy trials indicate that no substantial differences in effectiveness exist among second-generation antidepressants. | Conclusion is still valid and this portion of the CER does not need updating. | Good |
| Eighteen studies indicated no statistical differences in efficacy with respect to health-related quality of life (HRQOL). | | Consistent results from 18 trials indicate that the efficacy of second generation antidepressants with respect to quality of life does not differ among drugs. | Conclusion is still valid and this portion of the CER does not need updating. New data on adverse events (suggested by one expert) is covered in key question 4. | Good |
| Seven studies funded by the maker of mirtazapine reported that mirtazapine had a significantly faster onset of action than citalopram, fluoxetine, paroxetine, and sertraline. | | Consistent results from seven trials suggest that mirtazapine has a significantly faster onset of action than citalopram, fluoxetine, paroxetine, and sertraline. Whether this difference can be extrapolated to other second-generation antidepressants is unclear. Most other trials do not indicate a faster onset of action of one second-generation antidepressant compared with another. | Conclusion is still valid and this portion of the CER does not need updating. | Good |
| We identified no head-to-head trials for dysthymia. In placebo-controlled trials, significant differences in population characteristics make the evidence insufficient to identify differences between treatments. | | No head-to-head evidence exists. Results from five placebo controlled trials were insufficient to draw conclusions about comparative efficacy. No head-to-head evidence exists. One effectiveness trial provides mixed evidence about paroxetine versus placebo; patients older than 60 showed greater improvement on paroxetine; those younger than 50 did not show any difference. | Conclusion is still valid and this portion of the CER does not need updating. | Good |
| The only head-to-head evidence for treating patients with subsyndromal depression came from a nonrandomized, open-label trial comparing citalopram with sertraline. This study did not detect any differences in efficacy. Findings from two placebo-controlled trials were insufficient to draw any conclusions about the comparative efficacy and effectiveness. | | One nonrandomized, open-label trial did not detect any difference between citalopram and sertraline. Results from two placebo-controlled trials were insufficient to draw conclusions. | Conclusion is still valid and this portion of the CER does not need updating. | Good |
| **Key Question 1b. - If a patient has responded to one agent in the past, is that agent better than current alternatives at treating depressive symptoms?** | | **Key Question 1b. - If a patient has responded to one agent in the past, is that agent better than current alternatives at treating depressive symptoms?** |  |  |
| We did not find any efficacy evidence regarding this question. | | No evidence | Conclusion is still valid and this portion of the CER does not need updating. | Good |
|  | | **Key Question 1c. -Are there any differences in efficacy or effectiveness between immediate-release and extended-release formulations of second-generation antidepressants?** |  |  |
|  | | Results from two trials indicate that no differences in response to treatment exist between paroxetine IR and paroxetine CR. Two trials did not detect significant differences in maintenance of response and remission between fluoxetine daily and fluoxetine weekly. One trial reported higher response rates for venlafaxine XR than venlafaxine IR. |  | Not applicable. There was no similar KQ in the original report, and for this update the addition of this KQ yielded no important clinical differences |
| **Key Question 2a. - For adults with a depressive syndrome, do antidepressants differ in their efficacy or effectiveness for maintaining response or remission (i.e., preventing relapse or recurrence)?** | | **Key Question 2a. - For adults with a depressive syndrome that has responded to antidepressant treatment, do second-generation antidepressants differ in their efficacy or effectiveness for preventing relapse (i.e., continuation phase) or recurrence (i.e., maintenance phase) when a patient: o Continues the drug to which they initially responded, or o Switches to a different antidepressant?** |  |  |
| Three head-to-head RCTs suggest that no substantial differences exist between fluoxetine and sertraline, fluvoxamine and sertraline, and trazodone and venlafaxine, regarding relapse. Twenty-one placebo-controlled trials support the general efficacy and effectiveness of most second-generation antidepressants for preventing relapse or recurrence. No evidence exists for duloxetine. | | Based on results from six efficacy trials and one naturalistic study, no significant differences exist between escitalopram and desvenlafaxine, escitalopram and paroxetine, fluoxetine and sertraline, fluoxetine and venlafaxine, fluvoxamine and sertraline, and trazodone and venlafaxine for preventing relapse or recurrence. | Conclusion is possibly out of date and this portion of the CER may need updating to include evidence for duloxetine. | Fair. No duloxetine evidence ended up being included in this key question. |
| **Key Question 2b. - For adults receiving antidepressant treatment for a depressive syndrome that either has not responded (acute phase) or has relapsed (continuation phase) or recurred (maintenance phase), do alternative antidepressants differ in their efficacy or effectiveness?** | | **Key Question 2b. - For adults with a depressive syndrome that has not responded to acute antidepressant treatment or has relapsed (continuation phase) or recurred (maintenance phase), do alternative second-generation antidepressants differ in their efficacy or effectiveness?** |  |  |
| One head-to-head efficacy study and two effectiveness studies provide conflicting evidence on differences among second-generation antidepressants in treatment-resistant depression. The efficacy study suggests that venlafaxine is modestly more effective than paroxetine. A good-quality effectiveness study suggests that no substantial differences exist among bupropion SR, sertraline, and venlafaxine XR, but a fair-quality effectiveness study suggests that venlafaxine is modestly more effective than citalopram, fluoxetine, mirtazapine, paroxetine, and sertraline. | | Results from four trials suggest no differences or only modest differences between SSRIs and venlafaxine. Numerical trends favored venlafaxine over comparator drugs in three of these trials, but differences were statistically significant in only one trial, which compared venlafaxine with paroxetine. Results from two effectiveness studies are conflicting. Based on one trial rated good, no significant differences in effectiveness exist among bupropion SR, sertraline, and venlafaxine XR. One effectiveness trial found venlafaxine to be modestly superior to citalopram, fluoxetine, mirtazapine, paroxetine, and sertraline. | Conclusion is still valid and this portion of the CER does not need updating. | Good |
| **Key Question 3a. - Do medications differ in their efficacy and effectiveness in treating the depressive episode?** | | **Key Question 3 - In depressed patients with accompanying symptoms such as anxiety, insomnia, and neurovegetative symptoms, do medications or combinations of medications (including a tricyclic in combination with a second-generation antidepressant) differ in their efficacy or effectiveness for treating the depressive episode or for treating an accompanying symptoms?** |  |  |
| Antidepressant medications do not differ substantially in antidepressive efficacy for patients with MDD and anxiety symptoms. | | Results from five head-to-head trials suggest that efficacy does not differ substantially for treatment of depression in patients with accompanying anxiety. Results from eight head-to-head trials and three placebo-controlled trials suggest that no substantial differences in efficacy exist among second-generation antidepressants for treatment of accompanying anxiety symptoms. Results from one head-to-head study are insufficient to draw conclusions about the comparative efficacy for treating depression in patients with coexisting insomnia. Results from five head-to-head trials suggest that no substantial differences in efficacy exist among second-generation antidepressants for treatment of accompanying insomnia. Results are limited by study design; differences in outcomes are of unknown clinical significance. Results from one placebo-controlled trial of bupropion XL are insufficient to draw conclusions about treating depression in patients with coexisting low energy. Results from head-to-head trials are not available. Results from one placebo-controlled trial of bupropion XL are insufficient to draw conclusions about treating low energy in depressed patients. Results from head-to-head trials are not available. Results from two head-to-head trials are insufficient to draw conclusions about treating depression in patients with coexisting melancholia. Results are inconsistent across studies. | Conclusion is still valid and this portion of the CER does not need updating. | Good |
|  | | Results from two placebo-controlled trials are conflicting regarding the superiority of duloxetine over placebo. Results from head-to-head trials are not available. Evidence from one systematic review, two head-to-head trials (one poor) and five placebo-controlled trials indicate no difference in efficacy between paroxetine and duloxetine. Results from one head-to-head trial are insufficient to draw conclusions about the comparative efficacy for treating depression in patients with coexisting psychomotor change. Results from one head-to-head trial are insufficient to draw conclusions about the comparative efficacy for treating somatization in depressed patients. Results indicate similar improvement in somatization. Evidence from one open-label head-to-head trial is insufficient to draw conclusions about the comparative efficacy for treating coexisting somatization in depressed patients. Results indicate no difference in effectiveness. |  | Not applicable. This is a new conclusion not present in the original report, but is consistent with the overall "no difference in drugs" conclusion from the original report and thus would not change clinical practice. |
| **Key Question 3b. - Do medications differ in their efficacy and effectiveness in treating the accompanying symptoms?** | | **No Key Question 3b.** |  |  |
| One fair-quality head-to-head trial reported no statistically significant difference between fluoxetine and sertraline for treating depression in patients with psychomotor retardation. The same study found that sertraline was more efficacious than fluoxetine for treating depression in patients with psychomotor agitation. | | One fair-quality head-to-head trial reported no statistically significant difference between fluoxetine and sertraline for treating depression in patients with psychomotor retardation. The same study found that sertraline was more efficacious than fluoxetine for treating depression in patients with psychomotor agitation. | Conclusion is possibly out of date and this portion of the CER may need to be updated to add points regarding treatment of Parkinson’s symptoms and pain (see below). | Fair. The Parkinson's symptom data ended up not being included in the updated CER. |
| **Key Question 4 - For adults with a depressive syndrome, do commonly used antidepressants differ in safety, adverse events, or adherence? Adverse effects of interest include but are not limited to nausea, diarrhea, headache, tremor, daytime sedation, decreased libido, failure to achieve orgasm, nervousness, insomnia, and more severe events including suicide.** | | **Key Question 4a. - For adults with a depressive syndrome, do commonly used antidepressants differ in safety, adverse events, or adherence? Adverse effects of interest include but are not limited to nausea, diarrhea, headache, tremor, daytime sedation, decreased libido, failure to achieve orgasm, nervousness, insomnia, and more serious events including suicide.** |  |  |
| Constipation, diarrhea, dizziness, headache, insomnia, nausea, and somnolence were commonly and consistently reported adverse events. On average, 61 percent of patients in efficacy trials experienced at least one adverse event. Nausea and vomiting were found to be the most common reasons for discontinuation in efficacy studies. Overall, second-generation antidepressants have similar adverse events profiles. | | Adverse events profiles, based on 92 efficacy trials and 48 studies of experimental or observational design, are similar among second generation antidepressants. The incidence of specific adverse events differs across antidepressants. Meta-analysis of 15 studies indicates that venlafaxine has a higher rate of nausea and vomiting than SSRIs as a class. Results from seven trials indicate that mirtazapine leads to higher weight gains than citalopram, fluoxetine, paroxetine, and sertraline. Results from 15 studies indicate that sertraline has a higher incidence of diarrhea than bupropion, citalopram, fluoxetine, fluvoxamine, mirtazapine, nefazodone, paroxetine, and venlafaxine. Results from one systematic review confirm some of these findings. Results from six trials indicate that trazodone has a higher rate of somnolence than bupropion, fluoxetine, mirtazapine, paroxetine, and venlafaxine. | Conclusion is still valid and this portion of the CER does not need updating. | Good |
| Discontinuation syndromes (e.g., headache, dizziness, nausea) occurred in 0 to 86 percent of patients. Paroxetine and venlafaxine had the highest incidence of this problem, and fluoxetine the lowest incidence. | | A good systematic review indicates that paroxetine and venlafaxine have the highest rates of discontinuation syndrome; fluoxetine has the lowest. | Conclusion is possibly out of date and this portion may need updating. New analyses should be reviewed for methods, inclusion criteria, funding source. | Fair. Whatever new evidence was added did not change the conclusions |
| Overall discontinuation rates did not differ significantly between SSRIs as a class and bupropion, mirtazapine, nefazodone, trazodone, and venlafaxine. In the case of venlafaxine compared with SSRIs, higher discontinuation rates because of adverse events appear to be balanced by lower discontinuation rates because of lack of efficacy. | | Meta-analyses of numerous efficacy trials indicate that overall discontinuation rates are similar. Duloxetine and venlafaxine have a higher rate of discontinuations because of adverse events than SSRIs as a class. Venlafaxine has a lower rate of discontinuations because of lack of efficacy than SSRIs as a class. | Conclusion is possibly out of date and this portion may need updating based on new analysis showing lower drop out rate with escitalopram. | Fair. The escalitopram data did not end up in the conclusions. |
| Bupropion is associated with a lower incidence of sexual dysfunction than fluoxetine, paroxetine, and sertraline. In head-to-head trials, paroxetine consistently had higher rates of sexual dysfunction than comparators (fluoxetine, fluvoxamine, nefazodone, and sertraline). | | Results from six trials indicate that bupropion causes significantly less sexual dysfunction than escitalopram, fluoxetine, paroxetine, and sertraline. Among SSRIs, paroxetine has the highest rates of sexual dysfunction. | Conclusion is still valid and this portion of the CER does not need updating. | Good. |
| The existing evidence on the comparative risk for rare but severe adverse events, such as suicidality, seizures, cardiovascular events (i.e., elevated systolic and diastolic blood pressure and elevated pulse/heart rate), hyponatremia, hepatotoxicity, and serotonin syndrome, is insufficient to draw firm conclusions. | | No trials or observational studies assessing hyponatremia met criteria for inclusion in this review. One cohort study not meeting inclusion criteria suggested that hyponatremia was more common in elderly patients treated with various antidepressants than in placebo-treated patients. Evidence from existing studies is insufficient to draw conclusions about the comparative risk of hepatotoxicity. Weak evidence indicates that nefazodone might have an increased risk of hepatotoxicity. No trials or observational studies assessing serotonin syndrome were included in this review. Numerous case reports of this syndrome exist but were not included in this review. | Conclusion is possibly out of date and this portion of the CER may need updating based on new U.K. cohort study of over 200,000 patients. | Fair. The study identified by surveillance assessment, Rubino, A., N. Roskell, et al. (2007). "Risk of suicide during treatment with venlafaxine, citalopram, fluoxetine, and dothiepin: retrospective cohort study." BMJ 334(7587): 242 was not included in the updated CER. However, it seems to meet inclusion criteria of being an observational study of an adverse event with a sample size of greater than 1,000. |
|  | | Results from 11 observational studies (two good quality), five metaanalyses or systematic reviews (four good), and one systematic review yield conflicting information about the comparative risk of suicidality. Results from three studies (one good observational design) yield conflicting information about the comparative risk of seizures. Results from one good observational study and one pooled analysis yield noncomparative or conflicting information about the comparative risk of cardiovascular events. Evidence from existing studies is insufficient to draw conclusions about adherence in real-world settings. |  | This is a new conclusion, but consistent with the overall "no difference in drugs" and not one that will change clinical practice. |
| **No Key Question 4b.** | | **Key Question 4b. - Are there any differences in safety, adverse events, or adherence between immediate release and extended-release formulations of second-generation antidepressants?** |  |  |
|  | | Findings from one trial each indicate that no differences in harms exist between fluoxetine daily and fluoxetine weekly or between venlafaxine IR and venlafaxine XR. One trial provides evidence that paroxetine IR leads to higher rates of nausea than paroxetine CR. One trial provides evidence that fluoxetine weekly has better adherence rates than fluoxetine daily. Evidence from one observational study indicates that prescription refills are more common with the extended-release than the immediate-release formulation of bupropion. |  | This is a new conclusion, but consistent with the overall "no difference in drugs" and not one that will change clinical practice. |
| **Key Question 5 - How do the efficacy, effectiveness, or harms of treatment with antidepressants for a depressive syndrome differ for the following subpopulations:* Elderly or very elderly patients; *Other demographic groups (defined by age, ethnic or racial groups, and sex);* Patients with medical comorbidities (e.g., ischemic heart disease, cancer)?** | | **Key Question 5 - How do the efficacy, effectiveness, or harms of treatment with antidepressants for a depressive syndrome differ for the following subpopulations?**  **o Elderly or very elderly patients**  **o Other demographic groups (defined by age, ethnic or racial groups, and sex)**  **o Patients with medical comorbidities (e.g., ischemic heart disease, cancer)**  **o Patients with psychiatric and behavioral comorbidities (e.g., substance abuse disorders)**  **o Patients taking other medications** |  |  |
| No major differences in efficacy and effectiveness exist among second-generation antidepressants in elderly or very elderly populations. Indirect evidence suggests that efficacy among second-generation antidepressants does not differ between men and women. | | Evidence from 11 trials indicates that efficacy does not differ substantially among second-generation antidepressants for treating MDD in patients age 60 years or older. No head-to-head evidence found for dysthymia or subsyndromal depression. Results from one good placebo-controlled trial showed no difference between fluoxetine and placebo. No evidence in older patients with MDD. One effectiveness study showed greater improvement with paroxetine versus placebo in dysthymia patients older than 60 years; insufficient evidence to draw conclusions on comparative effectiveness. Results from six studies indicate that adverse events may differ somewhat across second-generation antidepressants in older adults. No head-to-head studies were found for dysthymia or subsyndromal depression. Two trials suggest differences between men and women in sexual side effects. Results from a subgroup analysis of one trial indicate significantly greater response with venlafaxine XR than fluoxetine in patients with MDD and comorbid generalized anxiety disorder. Placebo-controlled trials assessed efficacy in patients with the following comorbidities: alcohol/substance abuse, Alzheimer’s disease/dementia, arthritis, diabetes, HIV/AIDS, multiple sclerosis, stroke, and vascular disease. No head-to-head evidence exists on comparative efficacy. | Conclusion should be updated to include new data on racial/ethnic populations. | Fair. There were no changes to the conclusions or SOE for race or ethnicity subpopulations. |

| **Original Key Questions/Conclusions** | **Updated Key Questions/Conclusions (2011-2012)** | **2009 Prediction** | **Concordance** |
| --- | --- | --- | --- |
| **Comparative Effectiveness of Angiotensin-Converting Enzyme Inhibitors (ACEIs) and Angiotensin II Receptor Antagonists (ARBs) for Treating Essential Hypertension (Original report date - Nov 2007 and Update report date - Jun 2011)** | | | |
| **Key Question 1 - For adult patients with essential hypertension, how do ACEIs and ARBs differ in blood pressure control, cardiovascular risk reduction, cardiovascular events, quality of life, and other outcomes?** | **Key Question 1 - For adult patients with essential hypertension, how do ACEIs, ARBs, and direct renin inhibitors differ in blood pressure control, cardiovascular risk reduction, cardiovascular events, quality of life and other outcomes?** |  |  |
| ACEIs and ARBs appear to have similar long-term effects on blood pressure among individuals with essential hypertension. | ACEIs and ARBs appear to have similar long-term effects on blood pressure among individuals with essential hypertension. This conclusion is based on evidence from 77 studies (70 RCTs, 5 nonrandomized controlled clinical trials, 1 retrospective cohort study, and 1 case-control study) in which 26,170 patients receiving an ACEI or an ARB were followed for periods from 12 weeks to 5 years (median 24 weeks). Blood pressure outcomes were confounded by additional treatments and varying dose escalation protocols. Evidence concerning the effect of direct renin inhibitors on blood pressure is very limited and currently based on only three studies. These studies found the direct renin inhibitor to have a greater reduction in blood pressure compared to the ACEI ramipril (two studies) and no significant difference compared to the ARB losartan (one study). | Conclusion is still valid and this portion of the CER does not need updating. | Good |
| Due to insufficient numbers of deaths or major cardiovascular events in the included studies, it was not possible to discern any differential effect of ACEIs vs. ARBs for these critical outcomes. | Due to low numbers of deaths or major cardiovascular events reported, it was difficult to discern any differential effect of ACEIs versus ARBs versus direct renin inhibitors with any certainty for these critical outcomes. In 21 studies that reported mortality, MI, or clinical stroke as outcomes among 38,589 subjects, 38 deaths and 13 strokes were reported. This may reflect low event rates among otherwise healthy patients and relatively few studies with extended followup. Only 3 of these 21 studies (including 1 death) evaluated direct renin inhibitors versus ACEIs or ARBs, and therefore the evidence to discern any differential effects between these drug classes on mortality and major cardiovascular events was insufficient. | Conclusion is still valid and this portion of the CER does not need updating. | Good |
| No differences were found in measures of general quality of life; this is based on 4 studies, 2 of which did not provide quantitative data. | No differences were found between ACEIs and ARBs in measures of general quality of life; this is based on four studies, two of which did not provide quantitative data. No study evaluated the comparative effectiveness of direct renin inhibitors for quality-of-life outcomes. | Conclusion is still valid and this portion of the CER does not need updating. | Good |
| There was no statistically evident difference in the rate of treatment success based on use of a single antihypertensive, for ARBs compared to ACEIs. There were no consistent differential effects of ACEIs vs. ARBs on several potentially important clinical outcomes, including lipid levels, progression to type 2 diabetes mellitus, markers of carbohydrate metabolism/diabetes control, measures of LV mass or function, and progression of renal disease (either based on creatinine, GFR, or proteinuria). Relatively few studies assessed these outcomes over the long term. | There was no statistically evident difference in the rate of treatment success based on use of a single antihypertensive for ARBs compared to ACEIs. The trend toward less frequent addition of a second agent to an ARB was heavily influenced by retrospective cohort studies, where medication discontinuation rates were higher in ACEI-treated patients, and by RCTs with very loosely defined protocols for medication titration and switching. There were no relevant studies evaluating direct renin inhibitors. There were no consistent differential effects of ACEIs, ARBs, on several potentially important clinical outcomes, including lipid levels and markers of carbohydrate metabolism/diabetes control. There appears to be a small difference in change in renal function between ACEIs and ARBs (favoring ACEIs), but this difference is both small and most likely not clinically meaningful or significant. Relatively few studies assessed these outcomes over the long term. There were no studies that evaluated these outcomes in direct renin inhibitors. There was no evidence for an impact of ACEIs, ARBs, or direct renin inhibitors on glucose or A1c, and no included studies evaluated rates of progression to type 2 diabetes mellitus. Although we included 13 studies of LV mass/function, these were dominated by poor-quality studies with small sample sizes, and only one study included evaluation of a direct renin inhibitor. | Conclusion is still valid and this portion of the CER does not need updating. | Good |
| **Key Question 2 - For adult patients with essential hypertension, how do ACEIs and ARBs differ in safety, adverse events, tolerability, persistence, and adherence?** | **Key Question 2 - For adult patients with essential hypertension, how do ACEIs, ARBs, and renin inhibitors differ in safety, adverse events, tolerability, persistence with drug therapy, and treatment adherence?** |  |  |
| ACEIs have been consistently shown to be associated with slightly greater risk of cough than ARBs. There was no evidence of differences in rates of other commonly reported specific adverse events to quantify. | ACEIs have been consistently shown to be associated with greater risk of cough than ARBs (odds ratio 0.211; 95% CI0.159 to 0.281). For RCTs, this translates to a difference in rates of cough of 7.8 percent; however, for cohort studies with lower rates of cough, this translates to a difference of 1.2 percent. There were only two studies comparing direct renin inhibitors to ACEIs and these gave an estimated odds ratio of0.333 (95% CI of 0.2241 to 0.4933). The withdrawal rate for ARBs was found to have an estimated odds ratio of 0.565 (95% CI 0.453 to 0.704) compared with ACEIs. For RCTs, this translated to an absolute difference in withdrawals of 2.3 percent (5.4%versus 3.1%). The direct renin inhibitor trials did not find a statistically significant difference (odds ratio 0.886; 95% CI0.458 to 1.714) when compared with the withdrawal rate associated with ACEIs. There was no evidence of differences across treatments in rates of other commonly reported specific adverse events. | Conclusion is still valid and this portion of the CER does not need updating. | Good |
| Angioedema was reported only in patients treated with ACEIs; however, because angioedema was rarely explicitly reported in the included studies, it was not possible to estimate its frequency in this population. | Although several studies collected data on angioedema, the event rates were very low or zero for all studies; this limited our ability to accurately characterize the frequency of angioedema. In the four studies that did report episodes of angioedema, this adverse event was observed only in patients treated with an ACEI (five patients from three studies) or a direct renin inhibitor (one patient in one study). | Conclusion is still valid and this portion of the CER does not need updating. | Good |
| ACEIs and ARBs have similar rates of adherence based on pill counts; this result may not be applicable outside the clinical trial setting. Rates of continuation with therapy appear to be somewhat better with ARBs than with ACEIs; however, due to variability in definitions, limitations inherent in longitudinal cohort studies, and relatively small sample sizes for ARBs, the precise magnitude of this effect is unknown. | ACEIs and ARBs have similar rates of treatment adherence based on pill counts; this result may not be applicable outside the clinical trial setting. Rates of continuation with therapy appear to be somewhat better with ARBs than with ACEIs; however, due to variability in definitions, limitations inherent in longitudinal cohort studies, and relatively small sample sizes for ARBs, the precise magnitude of this effect is difficult to quantify. The three included studies evaluating direct renin inhibitors did not find evidence of differences in treatment adherence compared with ACEIs or ARBs. Persistence was not evaluated in any of the studies including direct renin inhibitors. | Conclusion is still valid and this portion of the CER does not need updating. | Good |
| **Key Question 3 - Are there subgroups of patients based on demographic characteristics (age, racial and ethnic groups, sex), use of other medications concurrently, or comorbidities for which ACEIs or ARBs are more effective, associated with fewer adverse events, or better tolerated?** | **Key Question 3 - Are there subgroups of patients—based on demographic and other characteristics (i.e., age, race, ethnicity, sex, comorbidities, concurrent use of other medications)—for whom ACEIs, ARBs, or direct renin inhibitors are more effective, are associated with fewer adverse events, or are better tolerated?** |  |  |
| Evidence does not support conclusions regarding the comparative effectiveness, adverse events, or tolerability of ACEIs and ARBs for any particular patient subgroup. | Evidence does not support conclusions regarding the comparative effectiveness, adverse events, or tolerability of ACEIs, ARBs, and direct renin inhibitors for any particular patient subgroup. | Conclusion is possibly out of date and this portion may need updating, based on new FDA data about possible adverse events. | Good |

| **Original Key Questions/Conclusions** | **Updated Key Questions/Conclusions (2011-2012)** | **2009 Prediction** | **Concordance** |
| --- | --- | --- | --- |
| **Comparative Effectiveness of Drug Therapy for Rheumatoid Arthritis and Psoriatic Arthritis in Adults (Original report date - Nov 2007 and Update report date - Jun 2012)** | | | |
| **Key Question 1 - For patients with rheumatoid arthritis or psoriatic arthritis, do drug therapies differ in their ability to reduce patient-reported symptoms, to slow or limit progression of radiographic joint damage, or to maintain remission?** | **Key Question 1 - For patients with PsA, do drug therapies differ in their ability to reduce disease activity, to slow or limit progression of radiographic joint damage, or to maintain remission?** |  | Note: in the update report the term "oral" was used to describe DMARDs previously described as "synthetic" |
| The data show no differences in radiographic outcomes over 2 years for leflunomide and methotrexate (MTX). One systematic review that included a meta-analysis of two RCTs suggested that higher proportions of patients on MTX than on leflunomide met the American College of Rheumatology (ACR) 20-percent improvement criteria at 1 year but statistical significance was lost at 2 years. | No head-to-head studies met inclusion criteria; unable to draw conclusions on the comparative efficacy of leflunomide and other treatments. Compared with placebo in one study, leflunomide produced better improvement in health-related quality of life and statistically significant, but not clinically significant, improvement in disease activity and functional capacity. No head-to-head studies met inclusion criteria; unable to draw conclusions on the comparative efficacy of MTX and other treatments. Current evidence was limited to placebo-controlled trials. Compared with placebo in one fair study, MTX resulted in greater improvement in physician assessment of disease activity than placebo. Existing comparative evidence did not support the superiority of one oral DMARD over another. Limitations to these trials included the wide range of MTX dosing in the trials.  Head-to-head trials showed no clinically important differences in efficacy among oral DMARD comparisons (methotrexate, sulfasalazine, leflunomide). The only head-to-head trial comparing biologic DMARDs (abatacept vs. infliximab) found no clinically important differences. | Conclusion is still valid and this portion of the CER does not need updating. | Good |
| In three studies, patients on etanercept had a faster onset of action than patients on infliximab, although no differences in effectiveness were apparent between the two agents. | No comparable conclusion in the update | Conclusion is still valid and this portion of the CER does not need updating. | Not applicable |
| Adjusted indirect comparisons indicate that anakinra has lower efficacy than anti-Tumor Necrosis Factor (TNF) drugs. | One systematic review of TNF inhibitors found that both TNF inhibitors and sulfasalazine are effective (similar withdrawals due to lack of efficacy); however, the data were insufficient to determine if the effect reached MCID. Less improvement in disease activity (ACR 52) for anakinra compared with etanercept and compared with adalimumab in MTC analyses in patients resistant to MTX. | Conclusion is still valid and this portion of the CER does not require updating. | Good |
| One prospective cohort study enrolled a population who failed initial RA treatment. After 12 months, patients on biologic Disease Modifying Antirheumatic Drugs (DMARDs) had almost four times higher odds of achieving functional independence percent and almost two times higher odds of achieving remission than patients on synthetic DMARDs. | In patients with early Ra, no clinically significant differences in clinical response between adalimumab or etanercept and MTX; in patients on biologic DMARDs, better radiographic outcomes than in patients on oral DMARDs. Faster improvement in quality of life with etanercept than MTX. | Conclusion is still valid and this portion of the CER does not need updating. | Good |
| Combination strategies of one or more synthetic DMARDs with corticosteroids have better outcomes than synthetic DMARD monotherapy. | Greater improvement in functional capacity for one oral DMARD plus prednisolone than for oral DMARD monotherapy. No difference in quality of life. | Conclusion is possibly out of date and this portion of the CER may need updating due to new studies which may change the strength of the conclusion. | Fair. The original report did not provide an SOE for this specific statement. The update report lists the SOE as moderate. |
| Overall, combination therapy of biologic DMARDs and MTX achieved better clinical response rates than monotherapies. | Better improvements in disease activity from combination therapy of biologic DMARDs (adalimumab, etanercept, infliximab, rituximab) plus MTX than from monotherapy with biologics. In MTX-naïve subjects with early aggressive RA, better ACR 50 responses, significantly greater clinical remission, and less radiographic progression in the combination therapy group. | Conclusion is out of date and this portion of the CER needs updating because of studies on new drugs and new studies of previously reviewed drugs which will not change the general conclusion but will extend it to new drugs and better refine the estimate of benefit. | Good. There are three new biologic DMARDs included in the update report. Certolizumab (a new DMARD) is mentioned prominently in some conclusions. |
| A combination of etanercept with sulfasalazine did not achieve better outcomes than etanerceptmonotherapy. | No comparable conclusion in the update | Conclusion is still valid and this portion of the CER does not need updating. | Not applicable |
| **Key Question 2 - For patients with rheumatoid arthritis or psoriatic arthritis, do drug therapies differ in their ability to improve functional capacity or quality of life?** | **Key Question 2 - For patients with PsA, do drug therapies differ in their ability to improve patient reported symptoms, functional capacity, or quality of life?** |  |  |
| Patients on MTX had less improvement in functional status and health-related quality of life than patients taking leflunomide. | Greater improvement in health-related quality of life (SF-36 physical component) for leflunomide | Conclusion is still valid and this portion of the CER does not need updating. | Good |
| Existing head-to-head evidence (three RCTs) supports no differences in efficacy between MTX and sulfasalazine by ACR 20,DAS, and functional capacity. | No head-to-head studies met inclusion criteria; unable to draw conclusions on the comparative efficacy or harms of sulfasalazine and other treatments. Current evidence was limited to placebo-controlled trials. Compared with placebo in one good systematic review study, sulfasalazine reduced disease activity. No differences for functional capacity. | Conclusion is still valid and this portion of the CER does not need updating. | Good |
| Greater improvements in functional capacity and quality of life were found with combination therapies (adalimumab, infliximab, or etanercept plus MTX) than with MTX alone. | In MTX-naïve subjects or those not recently on MTX, greater improvement in functional capacity and quality of life with combination therapy. In subjects with active RA despite treatment with MTX, no difference in functional capacity or quality of life. | Conclusion is possibly out of date and this portion of the CER may need updating, based on a new study about a previously included drug that had not been studied. | Fair. This conclusion did not change much although it is broadened from the original list of drugs. |
| **Key Question 3 - For patients with rheumatoid arthritis or psoriatic arthritis, do drug therapies differ in harms, tolerability, adherence, or adverse effects?** | **Key Question 3 - For patients with PsA, do drug therapies differ in harms, tolerability, adherence, or adverse effects?** |  |  |
| No differences in tolerability were reported for leflunomide, MTX, and sulfasalazine.  Discontinuation rates because of adverse events did not differ among leflunomide, MTX, or sulfasalazine. | Current evidence was limited to placebo controlled trials. Compared with placebo, leflunomide led to higher rates of withdrawals because of adverse events, diarrhea, and clinically significant increases in alanine aminotransferase. No consistent differences in tolerability and discontinuation rates. | Conclusion is still valid and this portion of the CER does not need updating. | Good |
| Biologic DMARDs were generally well tolerated in efficacy studies. Injection site reactions were substantially higher in patients using anakinra than in patients on adalimumab or etanercept. | The current evidence was limited to two cohort studies. Compared to anti-TNF monotherapy (adalimumab, etanercept, or infliximab), MTX plus anti-TNF produced similar disease activity response rates. Adjusted indirect comparisons found a more favorable withdrawal profile for certolizumabpegol than other biologic DMARDs. Also, etanercept and rituximab had a more favorable overall withdrawal profile than some other biologic DMARDs. Certolizumabpegol had fewer withdrawals due to lack of efficacy than adalimumab, anakinra, and infliximab. All but adalimumab, golimumab, and infliximab had fewer withdrawals than anakinra due to lack of efficacy. Both certolizumabpegol and infliximab had more withdrawals due to adverse events than etanercept and retuximab. Risks for injection site reactions apparently highest with anakinra. | Conclusion is possibly out of date and this portion of the CER may need updating, based on diversity of expert opinion | Good. New drugs and new results for harms. |
| Combination studies involving two synthetic DMARDs, including sulfasalazine and MTX, vs. one DMARD showed no differences in withdrawal rates because of adverse events. Combination studies including prednisone with one or more DMARDs also had no differences in discontinuation rates between groups. | No differences in discontinuation rates. | Conclusion is still valid and this portion of the CER does not need updating. | Good |
| Biologic combination vs. monotherapy: One RCT did not detect any synergistic effects of a combination treatment of etanercept and anakinra compared with etanerceptmonotherapy. The incidence of serious adverse events, however, was substantially higher with the combination treatment (14.8 percent vs. 2.5 percent; P = NR). | No differences in adverse events in efficacy studies. | Conclusion is possibly out of date and this portion of the CER may need updating based on diversity of reviewer opinion: one expert recalled studies that were not found on our targeted search. | Good. The conclusion changed as a result of more included studies. |
| A combination treatment of two biologic DMARDs can lead to substantially higher rates of severe adverse events than biologic DMARD monotherapy. The evidence, is limited to combinations of anakinra plus etanercept and abatacept plus anakinra, adalimumab, etanercept, or infliximab. | No head-to-head trials met inclusion criteria; unable to draw conclusions on the comparative efficacy of biologics and other treatments. Compared with placebo, adalimumab, etanercept, golimumab, and infliximab led to greater improvement in disease activity, functional capacity* and health-related quality of life.† Etanercept had a lower rate of withdrawals because of adverse events than infliximab in a prospective cohort study. Additional evidence was limited to placebo-controlled trials, where adverse events were not the primary outcome. Overall adverse event profiles appeared to be similar for biologic DMARDs and placebo. However, compared with placebo, we noted the following: adalimumab and etanercept had more injection-site reactions and adalimumab had fewer events of aggravated psoriasis than placebo Golimumab was associated with more malignancies than placebo in one RCT. Substantially higher rates of serious adverse events from combination of two biologic DMARDs than from monotherapy. | Conclusion is still valid and this portion of the CER does not need updating. | Good. The overall conclusion is unchanged but it now includes new biologic DMARDs. |
| No differences in adverse events were found between combinations of biologic and synthetic. | No differences in adverse events in efficacy studies. | This conclusion should possibly be reworded to make clear exactly what comparisons are being made. | Good |
| In general, no statistically significant differences in adverse events existed between combinations of biologic and synthetic DMARDs and synthetic DMARD monotherapy. Studies, however, were too small to assess reliably differences in rare but severe adverse events. An exception was a study with high-dose infliximab plus MTX therapy, which led to a statistically significantly higher rate of serious infections than MTX monotherapy. | Better tolerability profile for MTX plus abatacept, adalimumab, certolizumab, etanercept, and rituximab than for MTX monotherapy from meta-analysis. | Conclusion is probably out of date and this portion of the CER may need updating based on new data and diversity of expert opinion that might change the conclusion. | Good. New drugs and new adverse events results and change in conclusions. |
| **Key Question 4 - What are the comparative benefits and harms of drug therapies for rheumatoid arthritis and psoriatic arthritis in subgroups of patients based on stage of disease, history of prior therapy, demographics, concomitant therapies, or comorbidities?** | **Key Question 4 - What are the comparative benefits and harms of drug therapies for PsA in subgroups of patients based on stage of disease, history of prior therapy, demographics, concomitant therapies, or comorbidities?** |  |  |
| No comparative evidence exists on psoriatic arthritis (PsA) for any drugs. | No comparative evidence was identified for KQ 4. | Conclusion is still valid and this portion of the CER does not need updating. | Good |
| For RA the strength of evidence for age, sex, and comorbidities is very weak. | We found no studies that conducted comparisons by sex, race, or ethnicity, but we did include one fair systematic review that addressed age, one pooled analysis of RCTs, and a secondary database analysis of Medicare patients. One study directly compared the efficacy of etanercept in elderly RA patients (65 years of age or older) with younger RA patients (under 64years of age and older than 18) and found no significant difference in functional status between age groups. We identified two studies that addressed outcomes of RA patients with comorbidities. For RA patients with various high-risk conditions, one large placebo-controlled RCT of anakinra reported that there was no difference in serious adverse events or infections between the treated and placebo groups. A systematic review of 11 MTX trials of RA patients determined that those with renal impairment were directly at greater risk for experiencing MTX toxicity, and the greater the renal impairment the greater the toxicity effects. | Conclusion is still valid and this portion of the CER does not need updating. | Good. The update report continues to list this as a research priority. |
| A combination of either adalimumab plus MTX or infliximab plus methotrexate in patients with early, aggressive RA who were methotrexate naive led to better clinical and radiographic outcomes than MTX monotherapy. | No similar conclusion in the update | Conclusion is still valid and this portion of the CER does not need updating. | Not applicable |

| **Original Key Questions/Conclusions** | **Updated Key Questions/Conclusions (2011-2012)** | **2009 Prediction** | **Concordance** |
| --- | --- | --- | --- |
| **Comparative Effectiveness of Treatments to Prevent Fractures in Men and Women with Low Bone Density or Osteoporosis (Original report date - Dec 2007 and Update report date - Mar 2012)** | | | |
| **Key Question 1 - What are the comparative benefits in fracture reduction among and also within the following treatments for low bone density?** | **Key Question 1 - What are the Comparative Benefits in Fracture Risk Reduction Among the Following Therapeutic Modalities for low Bone Density: Bisphosphonates, Denosumab, Menopausal Hormone Therapy, Selective Estrogen Receptor Modulators (Raloxifene), Parathyroid Hormone, Calcium, Vitamin D, and Physical Activity?** |  |  |
| There is good evidence that alendronate, etidronate, ibandronate, risedronate, calcitonin, 1-34 PTH, and raloxifene prevent vertebral fractures | There is a high level of evidence from RCTs that alendronate, risedronate, ibandronate, zoledronic acid, denosumab, teriparatide, and raloxifene reduce the risk of vertebral fractures in postmenopausal women with osteoporosis. | Conclusion is still valid and this portion of the CER does not need updating. | Good |
| There is good evidence that risedronate and alendronate prevent both nonvertebral and hip fractures. | There is a high level of evidence from RCTs that alendronate, risedronate, zoledronic acid and denosumab reduce the risk of nonvertebral fractures in postmenopausal women with osteoporosis, and moderate evidence that teriparatide reduces the risk of nonvertebral fractures. There is a high level of evidence from RCTs that alendronate, risedronate, zoledronic acid, and denosumab reduce the risk of hip fractures in postmenopausal women with osteoporosis. | Conclusion is possibly out of date and this portion of the CER may need updating due to addition of ibandronate. | Fair. The ibandronate data did not end up changing this conclusion. |
| There is good evidence that zoledronic acid prevents vertebral and nonvertebral fractures and fair evidence that it prevents hip fractures. | There is a high level of evidence from RCTs that alendronate, risedronate, zoledronic acid, and denosumab reduce the risk of hip fractures in postmenopausal women with osteoporosis. | Conclusion is still valid and this portion of the CER does not need updating. | Good |
| There is evidence from one RCT that 1-34 PTH prevents nonvertebral fractures. | There is a high level of evidence from RCTs that alendronate, risedronate, zoledronic acid and denosumab reduce the risk of nonvertebral fractures in postmenopausal women with osteoporosis, and moderate evidence that teriparatide reduces the risk of nonvertebral fractures. | Conclusion is still valid and this portion of the CER does not need updating. | Good |
| There is good evidence that estrogen is associated w/ a reduced incidence of vertebral, nonvertebral, and hip fractures. | The original report found a high level of evidence that estrogen is associated with a reduced incidence of vertebral, nonvertebral, and hip fractures; however, studies identified for this report, which tended to focus on postmenopausal women with established osteoporosis (rather than on postmenopausal women with low bone density only or postmenopausal women in general) did not show significant reductions in fracture risk. | Conclusion is still valid and this portion of the CER does not need updating. | Fair. The differential effect based on severity is new information. |
| There are no data from RCTs on the effect of testosterone on prevention of fractures. | We also omitted several agents—etidronate, pamidronate, tamoxifen, and testosterone—based on their not being indicated or used for osteoporosis treatment, and also modified the question to include consideration of the sequential or combined use of different agents. | Conclusion is still valid and this portion of the CER does not need updating. | Not applicable as testosterone was deleted from the update |
| There is good evidence that there is no difference between calcium alone and placebo in preventing vertebral, nonvertebral, hip, and wrist fractures in postmenopausal women. | There is moderate evidence, based on a published systematic review and several RCTs, that there is no difference between calcium alone and placebo in reducing the risk for vertebral and nonvertebral fractures; however, calcium significantly reduced hip fracture risk in one pooled analysis, and overall fracture risk in another pooled analysis. The evidence is low, based on one head-to-head trial, that the combination of alendronate and calcium significantly decrease the risk for any type of clinical fracture compared with alendronate alone. | Conclusion is possibly out of date and this portion of the CER may need updating. The new meta-analysis should be assessed for quality, inclusion criteria, etc. | Fair. Individual studies did not make any difference in the conclusions |
| Vitamin D has varying effects on fracture prevention, depending on dose, analogs, and population. | A large body of literature showed mixed results for an effect of vitamin D in lowering the risk for fracture, varying with dose, fracture site, analogs (the various molecular and chemical forms of the vitamin, each of which has different biological activity), and population. Evidence is moderate that Vitamin D, 700 to 800 I.U. daily, particularly when given with calcium, reduces the risk of hip and nonvertebral fractures among institutionalized populations (one systematic review) and the overall risk of fractures (a second systematic review). The evidence is low, based on limited head-to-head trial data (two trials), for a difference in fracture incidence between menopausal hormone therapy and raloxifene or vitamin D. | Conclusion is still valid and this portion of the CER does not need updating. | Good |
| Based on limited data from head-to-head trials, superiority for the prevention of fractures has not been demonstrated for any agent within the bisphosphonate class. | The evidence is insufficient from head-to-head trials of bisphosphonates to prove or disprove any agent’s superiority for the prevention of fractures. | Conclusion is still valid and this portion of the CER does not need updating. | Good |
| Based on limited data from head-to-head trials, superiority for the prevention of vertebral fractures has not been demonstrated for bisphosphonates in comparison with calcitonin, calcium, or raloxifene. | There is a high level of evidence, based on six previously published systematic reviews, that there is no difference in vertebral, nonvertebral, or hip fracture risk with administration of vitamin D alone compared to administration of calcium alone. | Conclusion is still valid and this portion of the CER does not need updating. | Good |
| Based on six head-to-head RCTs, there was no difference in fracture incidence between bisphosphonates and estrogen. | Evidence is moderate, based on six head-to-head RCTs, that there is no difference in fracture incidence between bisphosphonates and menopausal hormone therapy. The evidence is insufficient from head-to-head trials of bisphosphonates to prove or disprove any agent’s superiority for the prevention of fractures. The evidence is insufficient, from three head-to-head trials of bisphosphonates compared to calcium, teriparatide, or raloxifene to prove or disprove superiority for the prevention of fractures. | Conclusion is still valid and this portion of the CER does not need updating. | Good |
| There are no data from RCTs on the effect on fracture prevention of exercise relative to the effect of agents used to treat or prevent osteoporosis. | The evidence is insufficient to low regarding the effect of physical activity on fracture risk, compared to placebo: One study showed a small effect on fracture prevention. No studies compared the effect of physical activity to that of other interventions. The evidence is insufficient regarding the use of combinations of osteoporosis therapies or sequential use of osteoporosis therapies in relation to fracture outcomes. |  | Good |
|  | Denosumab reduces the risk of vertebral, nonvertebral and hip fractures in postmenopausal women with osteoporosis. |  | This is a new conclusion about a new drug that was not in the original CER. |
| **Key Question 2 - How does fracture reduction resulting from treatments vary between individuals with different risks for fracture as determined by bone mineral density (borderline/low/severe), prior fractures (prevention vs. treatment), age, gender, glucocorticoid use, and other factors (e.g., community dwelling vs. institutionalized; vitamin D deficient vs. not)?** | **Key Question 2 - How Does Fracture Risk Reduction Resulting From Treatments Vary Between Individuals With Different Risks for Fracture as Determined by Bone Mineral Density, FRAX or Other Risk Assessment Score, Prior Fractures, age, sex, Race/Ethnicity and Glucocorticoid use, and Other Factors (e.g., Community Dwelling vs. Institutionalized, Vitamin D Deficient vs. not)?** |  |  |
| Alendronate, etidronate, ibandronate, risedronate, teriparatide, & raloxifene reduce the risk of fractures among high-risk groups, including postmenopausal women w/ osteoporosis. | Alendronate, ibandronate, risedronate, teriparatide, raloxifene, zoledronic acid, and denosumab reduce the risk of fractures among high risk groups including postmenopausal women with osteoporosis. | Conclusion is still valid and this portion of the CER does not need updating | Good |
| Calcitonin has been demonstrated to reduce the risk of fracture among postmenopausal women. | The original report included the peptide hormone calcitonin, but it has been excluded from this report at the subject matter experts’ request, since most authorities no longer consider calcitonin to be appropriate treatment for osteoporosis. | Conclusion is still valid and this portion of the CER does not need updating. | Not applicable as this drug was dropped the update. |
| Raloxifene prevents fractures in postmenopausal women at low risk for fracture. | Raloxifene prevents fractures in postmenopausal women at low risk for fracture as assessed by FRAX. | Conclusion is still valid and this portion of the CER does not need updating. | Good |
| The effect of estrogen on fracture prevention for women at low risk is uncertain. | No comparable conclusion in the update | Conclusion is still valid and this portion of the CER does not need updating. | Not applicable |
| Calcitonin, risedronate, and teriparatide reduce the risk of fracture among men. | Teriparatide and risedronate but not calcium and vitamin D reduce risk of fracture among men. | Conclusion is still valid and this portion of the CER does not need updating. | Good |
| In subjects treated with glucocorticoids, fracture risk reduction was demonstrated for risedronate and alendronate. | Among those treated with glucocorticoids, fracture risk reduction was demonstrated for risedronate and alendronate compared to placebo; and for teriparatide compared to alendronate. | Conclusion is still valid and this portion of the CER does not need updating. | Good |
| There is good evidence that tamoxifen does not prevent fractures among women at risk for breast cancer. | Although the original report included tamoxifen, it was excluded from this report, as it is not primarily used for osteoporosis prevention or treatment. | Conclusion is still valid and this portion of the CER does not need updating. | Not applicable |
| Reduction in fracture risk for subjects treated with alendronate, risedronate, or vitamin D has been demonstrated in populations at increased risk for fracture due to conditions that increase the risk of falling, including stroke with hemiplegia, Alzheimer's disease, and Parkinson's disease. | Reduction in fracture risk for subjects treated with alendronate, risedronate, or vitamin D has been demonstrated in populations at increased risk for fracture due to conditions that increase the risk of falling including stroke with hemiplegia, Alzheimer’s disease, and Parkinson’s. | Conclusion is still valid and this portion of the CER does not need updating. | Good |
| There are limited and inconclusive data on the effect of agents for the prevention and treatment of osteoporosis on transplant recipients and patients treated with chronic corticosteroids. | There are limited and inconclusive data on the effect of agents for the prevention and treatment of osteoporosis on transplant recipients and patients treated with chronic corticosteroids. |  | Good |
|  | In general, a high level of evidence suggests that bisphosphonates are at least as effective for older persons as for younger. Evidence is insufficient from trials assessing the effect of renal function on the efficacy of alendronate, raloxifene, and teriparatide. Two trials report no effect of renal function on the effects of these agents. However, in a third trial, impaired renal function reduced the efficacy. Raloxifene decreases the risk for vertebral fracture but not nonvertebral or hip fracture among postmenopausal Asian women, similar to other postmenopausal women. | lump | These are all new conclusions not presented in the original CER |
| **Key Question 3 - What are the adherence to and persistence with medications for the treatment and prevention of osteoporosis, the factors that affect adherence and persistence, and the effects of adherence and persistence on the risk of fractures?** | **Key Question 3 - Regarding Treatment Adherence and Persistence: a) What are the Adherence and Persistence to Medications for the Treatment and Prevention of Osteoporosis? b) What Factors Affect Adherence and Persistence? c) What are the Effects of Adherence and Persistence on the Risk of Fractures?** |  |  |
| Only 10 fracture trials reported rates of adherence to therapy. Five trials of calcium reported low rates of adherence. In two studies of daily oral bisphosphonates, more than 80 percent of patients took at least 70 percent of the drug. The other three trials reported high rates of adherence with risedronate therapy. |  | Conclusion is still valid and this portion of the CER does not need updating. | Good. While additional adherence studies were found their results were consistent with the findings from the original report. |
| There is evidence from 10 observational studies that real world adherence to therapy with alendronate, etidronate, risedronate, calcitonin, hormone replacement therapy (HRT), raloxifene, calcium, and vitamin D is poor among many postmenopausal women with osteoporosis. | Eighteen RCTs reported rates of adherence to therapy. Twelve trials with bisphosphonates and two trials with denosumab reported high levels of adherence (majority with over 90% adherence). Two trials with raloxifene had adherence rates 65-70%. Adherence rates are higher in clinical trials than in real life, likely reflecting the select populations and controlled environments in trials; in contrast, adherence rates in observational studies tend to resemble those in real life. | Conclusion is still valid and this portion of the CER does not need updating. | Good |
| There is evidence from one observational study that adherence to therapy with alendronate and risedronate is poor in many chronic glucocorticoid users. | There were no data specific to adherence in chronic glucocorticoid users in the update report. | Conclusion is still valid and this portion of the CER does not need updating. | Not applicable as there is no comparable conclusion in the update |
| There is evidence from 12 observational studies that persistence with therapy with alendronate, etidronate, risedronate, calcitonin, HRT, raloxifene, calcium, and vitamin D is poor in many men and postmenopausal women with osteoporosis. | There is evidence from 58 observational studies, including 24 using U.S. data, that adherence and persistence with therapy with bisphosphonates, calcium, and vitamin D is poor in many patients with osteoporosis. One study described adherence with teriparatide. No studies describe primary nonadherence (i.e. nonfulfillment). | Conclusion is still valid and this portion of the CER does not need updating. | Good |
| Based on evidence from observational studies, factors that affect adherence and persistence w/ medications include side effects of medications, absence of symptoms related to the underlying disease, comorbid conditions, ethnicity, socioeconomic status, & dosing regimens. Weekly users had higher persistence & adherence rates than daily users. In four observational studies comparing weekly and daily bisphosphonates, weekly users had higher persistence and adherence rates. | Definitions of adherence and persistence vary widely across studies and over time. The rates of adherence and persistence observed in the studies reviewed for this report reflect closely the rates seen and examined in prior systematic reviews on the topic, as well as in the previous report. Adherence and persistence as measured in observational studies is poor. In the U.S. studies overall, about half of patients appeared to show persistence with osteoporosis treatment at 1 year, with adherence ranging widely across studies. Based on 20 observational studies, dosing frequency appears to affect adherence/persistence: adherence is improved with weekly compared to daily regimens, but current evidence is lacking to show that monthly regimens improve adherence over that of weekly regimens. Based on evidence from 41 observational studies, many factors affect adherence and persistence with medications including, but not limited to, dosing frequency, side effects of medications, co-morbid conditions, knowledge about osteoporosis, and cost. Age, prior history of fracture, and concomitant medication use do not appear to have an independent association with adherence or persistence. | Conclusion is still valid and this portion of the CER does not need updating. | Good |
| There is evidence from one RCT that postmenopausal women who are nonadherent to treatment with calcium have a higher risk of fracture than women who are adherent to therapy. There is evidence from RCTs and observational studies that postmenopausal women who are nonadherent to treatment with alendronate, risedronate, HRT, calcium, or calcitonin have a higher risk of fracture than women who are adherent to therapy. There is evidence from one observational study that postmenopausal women w/ osteoporosis who are nonpersistent w/ alendronate and risedronate therapy have a higher risk of fracture than women persistent w/ these medications. | Evidence from a systematic review and 15 out of 17 observational studies suggest that decreased adherence to bisphosphonates is associated with an increased risk of fracture (vertebral, nonvertebral or both). | Conclusion is still valid and this portion of the CER does not need updating. | Good |
|  | The evidence on adherence to raloxifene, teriparatide, and other drugs and its association with fracture risk is insufficient to make conclusions. |  | Not applicable as there is no comparable conclusion in the update |
| **Key Question 4 - What are the short- and long-term harms (adverse effects) of the above therapies, and do these vary by any specific subpopulations?** | **Key Question 4 - What are the Short- and Long-Term Harms (Adverse Effects) of the Above Therapies (When Used Specifically To Treat or Prevent low Bone Density/Osteoporotic Fracture), and do These Vary by any Specific Subpopulations (e.g., the Subpopulations Identified in Key Question 2)?** |  |  |
| There is good evidence that there are no differences in the rates of serious cardiac events among bisphosphonates, calcium, vitamin D, calcitonin, PTH, and placebo. | The original report found no differences between alendronate (in two trials), ibandronate (in two trials), or risedronate (in one trial), and placebo in cardiac death; no studies were found for that report on zoledronic acid that reported cardiovascular deaths. For the present report, one new study on zoledronic acid, and one new study on risedronate74 found no differences (pooled OR for risedronate Inf+, 95% CI: 0.13, Inf+); and zoledronic acid (OR 0.61 95% CI: 0.26, 1.37). No studies were identified for the original or the current report that reported on these events with use of parathyroid hormone (PTH). The original or current report identified no trials of PTH that reported cardiac death. No studies identified for the original or the current report found any cases of acute coronary syndromes in trials of vitamin D or calcium. A new meta-analysis of 15 placebo-controlled trials of calcium (administered for bone health in all cases but one) identified a small but significant increase in the risk for myocardial infarction in pooled results of five trials that contributed patient-level data (HR 1.31, 95% CI: 1.02, 1.67, p=0.035).466 The pooled results of trial-level data showed a similar effect (pooled RR 1.27, 95% CI: 1.01, 1.59, p=0.038). However, a number of letters written in response to the review pointed out multiple concerns with the analyses that could have resulted in biased results. | Conclusion is still valid and this portion of the CER does not need updating. | Good |
| A significant increase in the risk of AF for zoledronic acid relative to placebo has been reported in one large RCT but not in another. A trend toward increased risk for alendronate relative to placebo has been reported in a single large RCT. | Evidence is insufficient regarding the risk for this event. The original report identified one study that showed a significant increase in the risk of atrial fibrillation for zoledronic acid relative to placebo but another that did not; the current report identified one additional trial that, when pooled with the two earlier trials of zoledronic acid, showed a significant increase in the risk for atrial fibrillation. A large Bayesian meta-analysis among users of bisphosphonates that did not reach statistical significance and several additional meta-analyses showed mixed results. In March 2010, the FDA issued a followup to its 2007 safety review, noting the inconsistency in the data and requesting that providers and patients report such side effects. Thus, a relationship between zoledronic acid and atrial fibrillation is unproven but still an area of active surveillance. | Conclusion is possibly out of date and this portion of the CER may need updating due to new evidence and difference in expert opinion. | Good. New evidence has diminished the likelihood this relationship is causal. |
| Relative to placebo, Raloxifene has an increased pooled risk for pulmonary embolism (PE), thromboembolic events, and mild cardiac events (including chest pain, palpitations, tachycardia, and vasodilation).Relative to placebo, the risk of PE for tamoxifen was elevated in one trial; the risk of thromboembolic events did not differ in this trial. | The original report identified two large studies that showed higher odds for PE among raloxifene participants than among placebo participants. The current report identified two additional studies that, when pooled with the original two, showed even higher risk for PE. Evidence is high for an increased risk for this event. | Conclusion is still valid and this portion of the CER does not need updating. | Good |
| In three placebo-controlled trials of estrogen that reported cerebrovascular accident, estrogen participants had higher odds than did participants who took a placebo. In the two trials that compared an estrogen-progestin combination with placebo, the combination participants had greater odds of stroke than did placebo patients. When four estrogen studies reporting thromboembolic events were pooled, estrogen participants had greater odds of reporting them than did placebo participants. Similar results were found when three studies comparing an estrogen-progestin combination with placebo were pooled. | Estrogen and estrogen-progestin combination participants had higher odds of cerebrovascular accident (CVA) and thromboembolic events than did placebo participants. | Conclusion is still valid and this portion of the CER does not need updating. | Good |
| Esophageal ulcerations were reported in trials of all the bisphosphonates except zoledronic acid. The only significant difference from placebo was found in one trial in which etidronate participants had higher odds of esophageal ulcers. | Regarding adverse events associated with the pharmacologic agents, raloxifene, estrogen, and combined estrogen-progestin increased the risk for thromboembolic events, and etidronate increased the risk for esophageal ulcerations and gastrointestinal perforations, ulcerations, and bleeding. | Conclusion is still valid and this portion of the CER does not need updating. | Good |
| Perforations, ulcerations, and bleeds (PUBs) were reported in trials of all the bisphosphonates except zoledronic acid. Etidronate participants had higher odds of PUBs than did placebo participants in three pooled studies. In two pooled trials of oral daily ibandronate, treated participants had lower odds of PUBs than did placebo participants. | Regarding adverse events associated with the pharmacologic agents, raloxifene, estrogen, and combined estrogen-progestin increased the risk for thromboembolic events, and etidronate increased the risk for esophageal ulcerations and gastrointestinal perforations, ulcerations, and bleeding. | Conclusion is still valid and this portion of the CER does not need updating. | Good |
| We categorized conditions such as acid reflux, esophageal irritation, nausea, vomiting, and heartburn as “mild upper gastrointestinal (GI) events.” Etidronate users had showed greater odds than for placebo participants. Pooled trials of pamidronate also showed greater odds for drug users than for placebo. Our pooled analyses found no difference between alendronate, ibandronate, risedronate, or zoledronic acid and placebo regarding mild upper GI events. | We categorized conditions such as acid reflux, esophageal irritation, nausea, vomiting, and heartburn as “mild upper GI events.” Pooled analysis of 50 studies of alendronate showed greater odds of all mild upper gastrointestinal (GI) events for alendronate than for placebo. In a head-to-head comparison of alendronate with denosumab, alendronate was also more strongly associated with mild upper GI events than was denosumab. Evidence is high regarding the risk for alendronate and mild upper GI events. | Conclusion is still valid and this portion of the CER does not need updating. | Good |
| In contrast, alendronate participants had higher odds of mild upper GI events than did etidronate participants in three pooled head-to-head trials. Alendronate participants also had higher odds of mild upper GI events in four head-to-head trials vs. calcitonin and four head-to-head trials vs. estrogen. Etidronate participants had higher odds of mild upper GI events in three head-to-head trials vs. estrogen. | We categorized conditions such as acid reflux, esophageal irritation, nausea, vomiting, and heartburn as “mild upper GI events.” Pooled analysis of 50 studies of alendronate showed greater odds of all mild upper gastrointestinal (GI) events for alendronate than for placebo. In a head-to-head comparison of alendronate with denosumab, alendronate was also more strongly associated with mild upper GI events than was denosumab. Evidence is high regarding the risk for alendronate and mild upper GI events. | Conclusion is still valid and this portion of the CER does not need updating. | Good |
| Risedronate participants had lower odds of musculoskeletal events than did placebo participants in nine pooled trials. In three pooled trials, zoledronic acid participants had higher odds of these events than did placebo participants. In two head-to-head trials, alendronate participants had greater odds of these events than did participants taking PTH. | In six trials of zoledronic acid, patients had 4.15 odds of myalgias, cramps, and limb pain compared to placebo. | Conclusion is still valid and this portion of the CER does not need updating. | Good |
| In five pooled trials of estrogen vs. placebo, estrogen participants had lower odds of breast cancer. Conversely, in three pooled studies of estrogen-progestin combination vs. placebo, treatment participants had higher odds of breast cancer. | Estrogen was not included in the update report | Conclusion is still valid and this portion of the CER does not need updating. | Not applicable |
| One estrogen-progestin study showed that treated participants had lower odds of colon cancer than did placebo participants. | Estrogen was not included in the update report | Conclusion is still valid and this portion of the CER does not need updating. | Not applicable |
| In three pooled studies of tamoxifen vs. placebo, tamoxifen participants had lower odds of breast cancer. Differences between raloxifene and placebo were not significant. | Tamoxifen was not included in the update report | Conclusion is out of date and this portion of the CER needs updating due to raloxifene being approved for breast cancer prevention. | Not applicable |
| Estrogen participants had more gynecological problems (such as uterine bleeding) than placebo participants. The same was true for users of estrogen-progestin combination in three pooled trials.In three pooled trials, tamoxifen participants had greater odds of gynecological problems than did placebo patients. | Estrogen was not included in the update report | Conclusion is still valid and this portion of the CER does not need updating. | Not applicable |
| Osteosarcoma was reported in only one study, a head-to-head trial of raloxifene vs. tamoxifen; differences between groups were not significant. | Comparisons with tamoxifen was not included in the update report | Conclusion is possibly out of date and this portion of the CER may need updating due to new evidence and difference in expert opinion. | Not applicable |
| There are no data from osteoporosis trials that describe an association between bisphosphonates and the development of osteonecrosis. In case reports and case series articles, we found many cases of osteonecrosis of the jaw in cancer patients taking intravenous bisphosphonates. Cases involved pamidronate, zoledronic acid, and alendronate. | One trial, one post hoc analysis of three trials, two large observational studies, and a review of 2,408 cases of osteonecrosis of the jaw in patients taking bisphosphonates for osteoporosis prevention or treatment found that the incidence of osteonecrosis of the jaw in this group was small, ranging from less than one to 28 cases per 100,000 person-years of treatment. | Conclusion is still valid and this portion of the CER does not need updating. | Good |
|  | Many potential barriers to adherence and persistence have been identified. Five of the most commonly assessed in published studies include age, prior history of fracture, dosing frequency, concomitant use of other medications, and adverse effects of the osteoporosis medications. The frequency with which these potential barriers appear in the literature does not necessarily correspond to their importance as barriers/factors related to adherence. |  | These are all new conclusions not presented in the original CER |
|  | Age, history of fracture, and number of concurrent medications do not appear to have an important independent association with adherence/persistence. |  | These are all new conclusions not presented in the original CER |
|  | Dosing frequency appears to affect adherence/persistence to a point: adherence is improved with weekly compared to daily regimens, but current evidence is lacking to show that monthly regimens improve adherence over that of weekly regimens. |  | These are all new conclusions not presented in the original CER |
|  | Adverse effects—and concerns about adverse effects—appear to be important predictors of adherence and persistence. Evidence from a systematic review and 15 out of 17 observational studies suggest that decreased adherence to bisphosphonates is associated with an increased risk of fracture (vertebral, nonvertebral or both). |  | These are all new conclusions not presented in the original CER |
|  | The evidence on adherence to raloxifene, teriparatide, and other drugs and its association with fracture risk is insufficient to make conclusions. |  | These are all new conclusions not presented in the original CER |
|  | Limited data from clinical trials and observational studies support a possible association between bisphosphonate use and atypical subtrochanteric fractures of the femur. Data are not consistent, nevertheless these data were sufficient for FDA to issue a Warning regarding this possible adverse event. |  | These are all new conclusions not presented in the original CER |
|  | Four observational studies that assessed whether the use of an oral bisphosphonate is associated with an increased risk of esophageal cancer had mixed findings. |  | These are all new conclusions not presented in the original CER |
| **No Key Question 5** | **Key Question 5 - With Regard to Treatment for Preventing Osteoporotic Fracture: a) How Often Should Patients be Monitored (via Measurement of Bone Mineral Density) During Therapy, how Does Bone Density Monitoring Predict Antifracture Benefits During Pharmacotherapy, and Does the Ability of Monitoring To Predict Antifracture Effects of a Particular Pharmacologic Agent Vary Among the Pharmacotherapies? b) How Does the Antifracture Benefit Vary With Long-term Continued use of Pharmacotherapy, and What are the Comparative Antifracture Effects of Continued Long-term Therapy With the Various Pharmacotherapies?** |  |  |
| **No Key Question 5a1** | **Key Question 5a1 - How Often Should Patients be Monitored via Measurement of Bone Mineral Density During Therapy?** |  |  |
| **No Key Question 5a2** | **Key Question 5a2 - How Does Bone Density Monitoring Predict Antifracture Benefits During Pharmacotherapy?** |  |  |
| **No Key Question 5a3** | **Key Question 5a3 - Does the Ability of Monitoring To Predict Antifracture Efficacy of a Particular Pharmacologic Agent Vary Among the Pharmacotherapies?** |  |  |
| **No Key Question 5b** | **Key Question 5b - How Does the Antifracture Benefit Vary With Long-term Continued use of Pharmacotherapy, and What are the Comparative Antifracture Efficacies of Continued Long-term Therapy With the Various Pharmacotherapies?** |  |  |
|  | No evidence exists from RCTs regarding how often patients’ BMD should be monitored during osteoporosis therapy. |  | These are all new conclusions not presented in the original CER |
|  | A high level of evidence exists from RCTs that lumbar spine and femoral neck BMD changes from serial monitoring predict only a small percentage of the change or do not predict the change in fracture risk from treatment with antiresorptives, including alendronate, risedronate, raloxifene, and teriparatide. |  |  |
|  | In RCTs, even people who lose BMD during antiresorptive therapy benefit from a substantial reduction in risk of vertebral fracture. Greater increases in BMD did not necessarily predict greater decreases in fracture risk. Thus, improvement in spine bone mineral density during treatment with currently available osteoporosis medications accounts for a predictable but small part of the observed reduction in the risk of vertebral fracture. Vertebral fracture risk is reduced in women who lose femoral neck BMD with teriparatide treatment. Evidence is high for this conclusion. |  |  |
|  | Evidence is moderate (one large RCT) that, compared to using alendronate for 5 years followed by discontinuation after 5 years, continuous use of alendronate for 10 years resulted in a lower risk of vertebral fracture. |  |  |

| **Original Key Questions/Conclusions** | **Updated Key Questions/Conclusions (2011-2013)** | **2009 Prediction** | **Concordance** |
| --- | --- | --- | --- |
| **Comparative Effectiveness of Epoetin and Darbepoetin for Managing Anemia in Patients Undergoing Cancer Treatment (Original report date - May 2006 and Update report date - April 2013)** | | | |
| **Key Question 1 - What are the comparative efficacy and safety of epoetin (alfa or beta) and darbepoetin? Outcomes of interest include hematologic responses, transfusions, tumor response to therapy, overall survival, quality of life, thromboembolic complications, and other adverse events.** | **Key Question 1 (KQ1). What are the comparative benefits and harms of erythropoiesis-stimulating agent (ESA) strategies and non-ESA strategies to manage anemia in patients undergoing chemotherapy or radiation for malignancy (excluding myelodysplastic syndrome and acute leukemia)?** |  |  |
| The evidence does not show any clinically significant difference between epoetin and darbepoetin in hemoglobin response, transfusion reduction, and thromboembolic events (TEE). For each of the above outcomes, more evidence is available on epoetin than darbepoetin. | ESAs reduced the proportion of patients receiving transfusions (overall strength of evidence moderate) without meaningful difference between epoetin and darbepoetin (overall strength of evidence moderate). Table A shows data on transfusion risk. There is a consistent body of evidence, although somewhat limited by trial quality, that ESAs reduce the probability of transfusion in the setting of cancer treatment. These agents do not eliminate the chance of receiving transfusions. | Conclusion is possibly out of date and this portion of the CER may need updating, based on new data and difference in expert opinion. | Fair. New data did not change the conclusion about equivalence of epoetin and darbepoetin. |
| The evidence is not sufficient for conclusions on effects of either epoetin or darbepoetin on quality of life (QoL), tumor response and progression, survival, or adverse outcomes other than TEE.Trials did not completely or consistently report quality of life (QoL) results, so 12 potentially relevant studies were unusable for this analysis, and quantitative analysis could not be performed for the 15 remaining studies. Overall, QoL measures tended to favor treatment with epoetin or darbepoetin. | ESAs did not affect survival over the longest available followup (overall strength of evidence low). Treating to high target hemoglobin levels (greater than 12 g/dL) was accompanied by improved health-related quality of life (HRQoL) scores (e.g., the Functional Assessment of Cancer Therapy [FACT] Fatigue score; overall strength of evidence low). Any clinical significance of the improvement in HRQoL is likely to be small. On average, the difference in change between treatment arms was less than the estimated minimal clinically important difference (a value of 3 for the FACT-Fatigue score). | Conclusion is still valid and this portion of the CER does not need updating. | Good. Changes noted in update were considered "small". |
| The limited evidence available does not suggest that erythropoietic stimulants improve solid tumor response to a concurrent course of cancer therapy. Whether erythropoietic stimulants accelerate progression of some cancers, as reported by one study is uncertain. | 22 trials reported some outcome related to survival with disease progression; 3 reported significant differences in disease-free or progression-free survival, one trial in favor of epoetin and two in favor of control | Conclusion is out of date and this portion of the CER needs updating based on new data and agreement of expert opinion. | Good. Major change in conclusions about safety. There are now black box warnings about this risk. The black box warning describes the results of two additional studies showing increased mortality and more rapid tumor progression in patients with non advanced breast and cervical cancers when dosed to target Hb of ≥12 g/dL. |
| Of 40 RCTs reporting on survival, only seven were actually designed to assess effects on survival. No studies designed to test survival used epoetin or darbepoetin as currently recommended; rather, all seven trials sought to maintain Hb levels >12 g/dL. Two of the seven trials, one on metastatic breast cancer (n=939) and one on head and neck cancer (n=351), showed poorer overall survival for patients treated with epoetin; this prompted an FDA safety review in May 2004 and revised product labeling to indicate that clinicians should avoid targeting Hb concentrations above 12 g/dL. Of the other five trials, survival appeared poorer with erythropoietic stimulant in three and better in two, but most results were not statistically significant. Analysis of mortality in all 40 trials shows no overall benefit of darbepoetin or epoetin on survival. Neither higher than recommended target Hb nor any other single patient- or treatment-related factor explained why some trials showed a detriment in survival and others did not. | ESAs increased mortality during and shortly following treatment (in this review, referred to as “on-study mortality”; overall strength of evidence moderate). Table C shows on-study mortality data. ESAs increased mortality during the active treatment or “on-study period” (median study duration 3 months) without apparent difference between epoetin and darbepoetin. There was one additional death for every 59 treated patients when the control arm on-study mortality was 10 percent, and there was one additional death for every 588 treated patients when the control armon-study mortality was 1 percent. While there was no discernible increase in mortality with ESA use over the longest available followup, many trials did not include an overall survival endpoint and potential time-dependent confounding was not considered. | Conclusion is probably out of date and this portion of the CER may need updating based on new data and the majority of expert opinion. | Good. Major change in conclusions about safety. There are now black box warnings about this risk. The black box warning describes that data are not sufficient to exclude the possibility of shortened survival and tumor progression in patients with cancer when ESAs are dosed to reach aHb level between 10 and 12 g/dL. Added information that ESAs caused tumor growth and shortened survival in patients with advanced breast, head and neck, lymphoid, and non–small-cell lung cancer when they received a dose that attempted to achieve Hb ≥12 g/dL. |
| For other adverse events, reporting is incomplete, representing less than one-third of patients. Studies did not use consistent definitions of events and severity. Overall, adverse events were more frequent with epoetin or darbepoetin than control, but pooled results did not show statistically significant differences. | ESA treatment increased the risk of thromboembolic events (overall strength of evidence moderate). Epoetin and darbepoetin conferred similar risks. | Conclusion is possibly out date and this portion of the CER may need updating based on differing expert opinion. | Good |
| **Key Question 2 - How do alternative dosing strategies affect the comparative efficacy and safety of epoetin and darbepoetin?** | **KQ2: How do alternative thresholds for initiating treatment compare as regards their effect on the benefits and harms of erythropoietic stimulants?** [An update of KQ2 was not recommended— “How do alternative dosing strategies affect the comparative efficacy and safety of epoetin (alfa or beta) and darbepoetin?” and the TEC EPC concurred. It is not included in the current Update KQs.] |  |  |
| For each of the following pairs of dosing strategies, one large trial reported no statistically significant difference between strategies: fixed-dose compared to dose based on weight, one trial each for epoetin and darbepoetin; fixed-dose epoetin administered weekly vs. thrice weekly; fixed dose epoetin administered weekly vs. every 3 weeks; and darbepoetin using an initial loading dose versus constant weight-based dosing regimens. The remaining 14 trials were too small to interpret. |  | Conclusion is still valid and this portion of the CER does not need updating. | Not applicable . No update of this KQ was done for the report. |
| **Key Question 3 - How do alternative thresholds for initiating treatment or alternative criteria for discontinuing therapy or duration of therapy affect the efficacy and safety of erythropoietic stimulants?** | **KQ3: How do different criteria for discontinuing therapy or for optimal duration of therapy compare as regards their effect on the benefits and harms of erythropoietic stimulants?** |  |  |
| Three unblinded randomized trials, not yet published, compared using erythropoietic stimulant therapy soon after mild anemia developed vs. delaying treatment until Hb had fallen below a predefined threshold of moderate anemia. Comparisons were ~11 g/dL vs. 9 g/dL; ~11 g/dL vs. 10 g/dL; and ~13 g/dL vs. 10 g/dL. All patients in the mild anemia arms were treated with an erythropoietic stimulant; of patients in whom treatment was delayed until moderate anemia developed, 19 percent, 63 percent, and 44 percent, respectively, were treated with erythropoietic stimulant. Transfusion was more frequent when treatment was delayed until moderate anemia developed, but the difference was not statistically significant in any study. One trial reported a statistically significant increase in TEE among patients who were treated for mild anemia compared with those who were treated for moderate anemia. | No randomized controlled trials were identified that fulfilled the review’s inclusion criteria for studies of discontinuing therapy or defining optimal duration of therapy. | Conclusion is probably out of date and this portion of the CER may need updating based on new evidence and the majority of expert opinion. | Good. Change in evidence from "three trials…" to "no trials…" |
|  | ESAs reduce the need for transfusions and increase the risk of thromboembolism. A detectable relative increase in mortality risk, which is higher with lower underlying absolute mortality risk, accompanies their use. An individual patient receiving ESAs will have, on average, better quality-of-life FACT-Fatigue scores, but of a magnitude less than the minimal clinically important difference. In a cohort decision model in which increased hemoglobin determined the utility-based measure of improvement in quality of life, ESAs were accompanied by some additional expected quality-adjusted life-years—consistent with the small difference in FACT-Fatigue scores. However, expected life-years were always lost, and the loss was greater with higher underlying absolute mortality risk. |  | This is a new conclusion and reflects a change in safety. |
| **Key Question 4 - Are any patient characteristics at baseline or early hematologic changes useful to select patients or predict responses to treatment with erythropoietic stimulants? The outcome of interest is limited to hematologic response.** | **There is no KQ 4 for the updated report: An update of KQ4 was not recommended, “Are any patient characteristics at baseline or early hematologic changes useful to select patients or to predict responses to treatment with erythropoietin?” This recommendation was based on expert opinion that referred to patient treatment characteristics and FDA labeling. However, the BCBSA TEC EPC judged that updating this question would be of little value. The literature reviewed in the 2006 CER was related to single or multifactorial algorithmic predictive testing. None was promising, and the literature has no bearing on the FDA changes to labeling, which are closely tied to the evidence for KQ1. This question was not included in the update.** |  |  |
| Available evidence does not identify any single patient factor as clinically useful to guide treatment decisions. Potential predictive factors, measured at baseline (e.g., serum erythropoietin level or observed/predicted ratio [O/P ratio], serum ferritin) or early after starting treatment (e.g., Hb increase, serum ferritin, reticulocyte increase), were found to have either weak ability or no ability to discriminate between responders and nonresponders. Seven algorithms combining multiple factors, potentially more useful to predict Hb response, are each currently supported only by one study. The largest of these studies do not report sufficient predictive ability for any algorithm to establish clinical utility for selecting treatment. |  | Conclusion is possibly out of date and this portion of the CER may need updating, based on differing expert opinion. | Not applicable as this KQ was not updated in the CER |
